# A preliminary study on the strand stress of transmission line in tension setting out

**DOI:** 10.1038/s41598-022-13300-3

**Published:** 2022-06-08

**Authors:** Xin Hu, Qingming Zhang, Zezhong Zhao, Cheng Shang, Xiaoming Rui

**Affiliations:** 1 State Key Laboratory of Explosion Science and Technology, Beijing Institute of Technology, Beijing, 100081 China; 2 School of Energy Power and Mechanical Engineering, North China Electric Power University, Beijing, 102206 China

**Keywords:** Civil engineering, Energy infrastructure, Mechanical engineering, Energy infrastructure

## Abstract

Transmission lines often suffer from conductor damage during operation, most of which are caused by excessive local stress of aluminum strands. However, one of the causes of conductor damage may be the tension paying off passing the pulley. At the same time, there are relatively few researches on the damage of conductor causing by passing pulley. Therefore, this paper studies the inter strand stress characteristics of aluminum strand when tension paying off passes through the pulley. The influence of envelope angle, tension load and friction on the stress characteristics between aluminum strands is studied by numerical simulation. The results show that the equivalent stress of the neutral layer of the aluminum strand is small, and the maximum equivalent stress appears at the contact position of the adjacent strand. The larger the envelope angle and tension load of the aluminum strands, the greater the equivalent stress in the cross section. Furthermore, the friction between aluminum strands has a certain effect on reducing the equivalent stress in the cross section. The equivalent stress of the aluminum strand increases from the outer layer to the inner layer, so the inner aluminum strand is more likely to be damaged than the outer layer. Finally, the experiment and the simulation have both been carried on, and then it is shown that the stress value in the corresponding section has a good consistency.

## Introduction

The normal and stable operation of overhead transmission lines is the most important part of the line engineering. It is fairly important to reduce the damage of conductor and the fault of transmission line in the safety protection of transmission line^1–5^. However, in the process of transmission line operation, conductor damage often occurs. Most of the cases show that the aluminum strand is under the action of multiple loads, which causes the local stress of the aluminum strand to be too large, resulting in the damage and fracture of the conductor^6–9^. However, the damage of the conductor may also be due to the large local stress that has been generated during the construction and setting out of the conductor, or even the partial damage of the aluminum strand. At present, most of the researches on conductor damage are focused on the conductor running under complex working conditions, while the researches on conductor damage in tension paying off construction, especially in the process of passing pulley, are less, and the researches on relevant conductor models and mechanical theories are relatively weak^10,11^. Therefore, it is very necessary to study the influence of tension setting out on the conductor. At the same time, this study is of great significance for power workers to protect the transmission conductor^12^.

Researchers all over the world have begun to study the construction technology of tension setting out and the stress characteristics of conductor for a long time^13–15^. At present, the relevant technology is relatively advanced. At the same time, there are many research methods, such as the theoretical reasoning proof, the finite element simulation analysis of mathematical model, and the comparison of the specific test verification, etc. Zhou et al.^16^ studied the relationship between the size of the bottom of the slot and the wear of the conductor, and put forward some useful suggestions for the size of the bottom of the slot of the stringing pulley. Raoof et al.^17^ proposed the main characteristics and some new progress of the real multi-layer structure steel strand analysis model. These results show the indirect contact forces among strands, layers and the relative displacements of strands in the spiral structure with fixed ends. Nawrocki et al.^18^ established the model of wire rope strand by using relevant finite element software, and applied axial force and axial and torsional combined load to study the stress characteristics of each layer of wire rope and between strands. Sarma et al.^19^ pointed out that the transmission line will generate corona, and will produce electric field effect, radio interference and other situations on the surrounding areas of the line, thus causing environmental damage. Papailiou et al.^20^ proposed a new type of conductor model to study the mechanical characteristics of overhead conductors. The model considered the friction and the slip between the strands in the bending process, derived the relevant calculation formula, and verified the correctness of the model through relevant tests. Kenta^21^ extended the research based on Papailiou K. et al. considering the transition process from adhesion state to slip state between stranding layers, the pure bending process of cable was simulated and tested without axial tension. In order to obtain the influencing factors of the variation of the bending stiffness of the conductor, Through the methods of finite element simulation, theoretical calculation and experimental measurement, the bending stiffness of the conductor under the tension load has been analyzed detailedly by Yang et al.^22–25^. Taking 1660mm^2^ large section carbon fiber composite core conductor as the research object, Wan et al.^26,27^ completed the theoretical calculation method of internal stress when the conductor passes through the pulley, and calculated the internal stress when the 1660mm^2^ large section carbon fiber composite core conductor passes through the pulley with different bottom diameters.

Based on the above researches, some extraordinary theoretical achievements and experience haced been achieved in transmission line construction, tension setting out technology and conductor stress characteristics obviously. However, the problem of conductor damage during the tension paying off construction is still unsolved perfectly. At the same time, investigation mostly on the stress characteristics of the conductor are based on the model of the whole conductor, which cannot clearly analyze the inter strand stress state inside the conductor. Therefore, through a relatively simplified conductor model, ABAQUS numerical simulation software is implemented perfectly to analyze the mechanical behavior of the conductor passing the pulley during the tension paying off process, and then studies the inter strand stress characteristics of the aluminum strand in the bending state. Then, the stress of the aluminum strand under the bending state is analyzed by use of simulating the situation of the conductor passing the pulley again through the test, so as to provide certain theoretical basis for the tension paying off construction.

## Theoretical analysis and model construction of conductor passing pulley

### Relevant theoretical analysis

#### Analysis of stress process of conductor passing through pulley

In the tension paying off construction, the conductor will pass through the paying off block at each tower. As the pulley induces extrusion and bending of the conductor, bending deformation and local stress can be stimulated, so the conductor passing through the block is a relatively vulnerable process of aluminum strand in the tension paying off construction. Therefore, when focusing on the stress characteristics of the transmission line in tension setting out, it is necessary to analyze the stress process of the conductor passing the pulley firstly.

**Figure 1:**
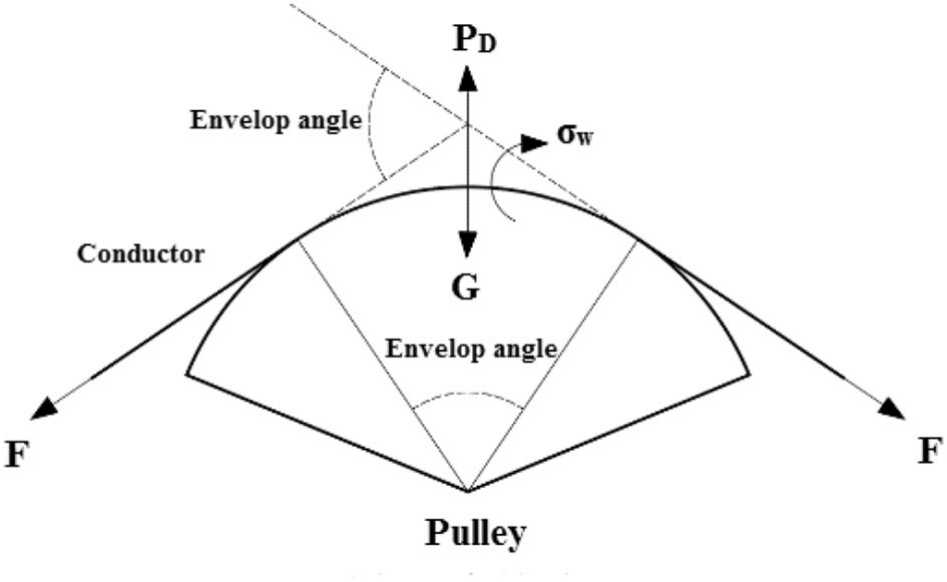
Force diagram of conductor passing through pulley.

When the conductor is setting out in tension, the tension load will not exceed 16% RTS of the rated conductor and the envelope angle between the conductor and the pulley generally will not exceed by 30. According to this working condition, the mechanical analysis of the conductor passing the pulley is carried out, as shown in Fig. 1 below.

In Fig. 1, the conductor will be exerted by three kinds of loads during tension paying off and passing the pulley:

1. *P*_*D*_ is the pressure of pulley on conductor. The following formula can be used for calculation:
  1 $$ P_{D} = \frac{G}{R\alpha B} $$
2. *F* is the load on the conductor in axial direction during tension setting out. The tensile stress in the axial direction can be obtained as follows:
  2 $$ \sigma_{L} = F/A $$
3. σ_w_ is the bending stress of the strand when the conductor passes through the pulley. It can be expressed by Bach formula^28^:
  3 $$ \sigma_{w} = K_{1} \frac{\delta }{{D_{S} }}E $$

In these equations, *G*(N) is the gravity of conductor on pulley; *R*(mm) describes the bottom radius of pulley; α(°) denotes the envelope angle of conductor passing through pulley; *B*(mm) expresses the contact width between conductor and pulley, and we usually take one third of the conductor diameter; *σ*_*L*_(MPa) is the tensile stress of conductor in axial direction; The section area of aluminum strand is defined by *A*(mm^2^); *δ*(mm) is the diameter of single aluminum strand of conductor; *K*_*1*_ is the characteristic coefficient of bending stress and we could take the empirical coefficient *K*_*1*_ = 0.375; *E* is the elastic modulus of aluminum; *D*_*S*_(mm) is the calculated value of pulley groove bottom diameter.

#### Stress Calculation of Strand Section in Bending State

**Figure 2:**
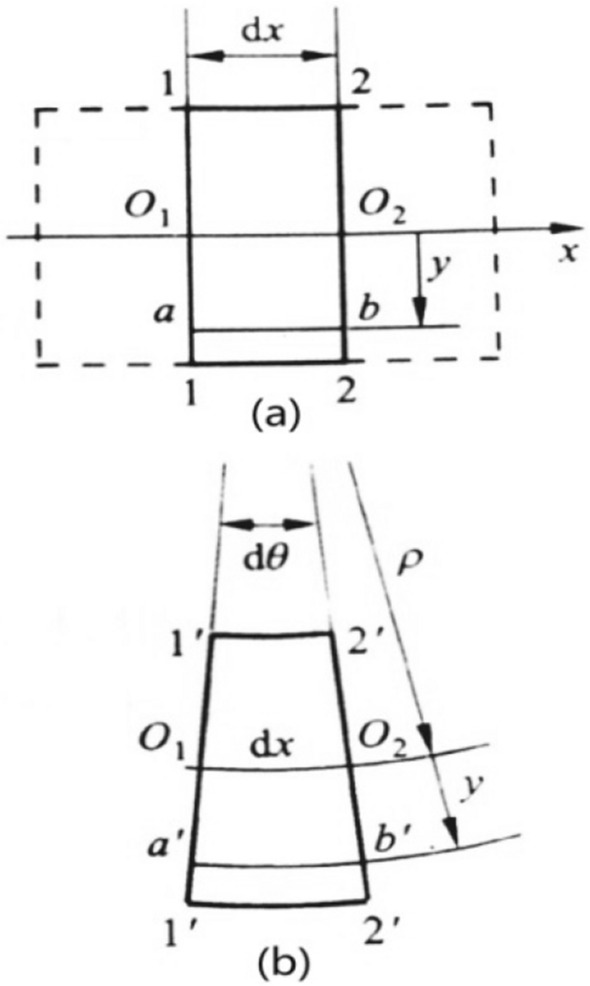
Diagram of pure bending geometric deformation of micro element beam.

It is assumed that there is no prestress on the strand section while the ACSR is not subjected to external force. As shown in Fig. 2, a micro element segment is cut from the beam subject to pure bending, and the coordinate axes *y* and *x* are respectively established along the longitudinal symmetry axis and neutral axis of the section. After the beam is bent, the straight line *ab* becomes the arc *a’b’*. If the relative angle between Sect. 1-1 and Sect. 2-2 is *dθ*, and the radius of curvature of the neutral layer is *ρ*, then the normal strain of straight line *ab* can be obtained.

4 $$ \varepsilon = \frac{{l_{{db^{^{\prime}} }} }}{dx} = \frac{(\rho + y)d\theta - \rho d\theta }{{\rho d\theta }} = \frac{y}{\rho } $$

Based on Hooke's law, we can get the normal stress at the distance y from the neutral axis on the cross section

5 $$ \sigma = E\frac{y}{\rho } $$

**Figure 3:**
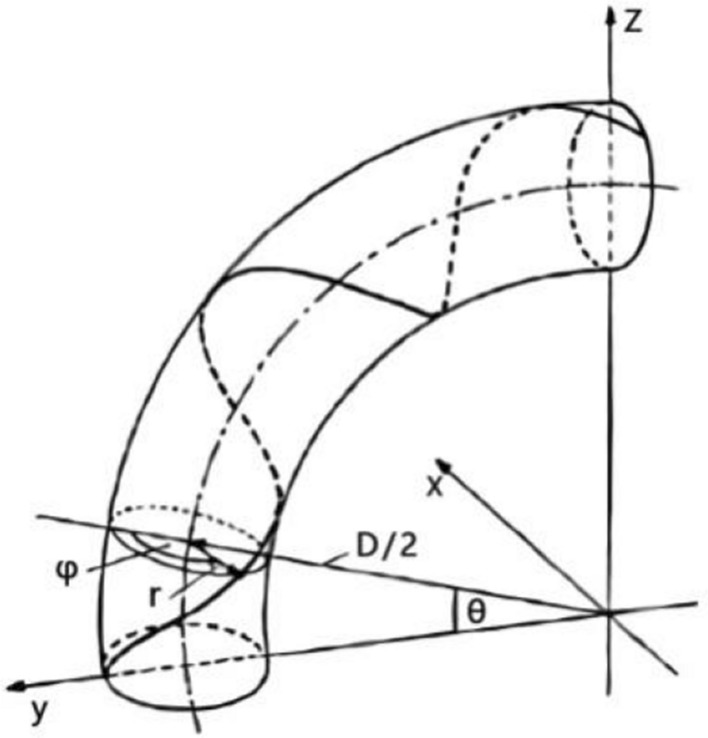
Diagram of conductor bending.

**Figure 4:**
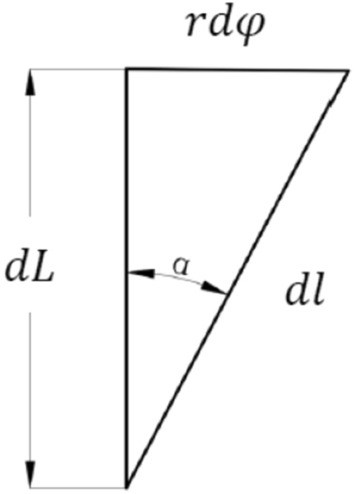
Axis increment diagram of strand.

Similarly, supposing that the radius of curvature is known at the section of the strand when the strand is subjected to bending load, the corresponding normal stress can be calculated according to the above equation. Figure 3 describes the spatial model of a strand of conductor in bending state. where *r* is the radius of the stranding layer where the strand is located, *D* is the curvature diameter of the strand, *φ* is the angle between the line between the section center of the strand and the section center of the strand and the curvature radius of the strand, and *θ* is the angle between the curvature radius and the y-axis in the starting plane of the strand. Then we can get the parameter equation of the bobbin as follows:

6 $$ \left\{ \begin{gathered} x = - r\sin \varphi \hfill \\ y = \frac{D}{2}\cos \theta + r\cos \varphi \cos \theta \hfill \\ z = \frac{D}{2}\sin \theta + r\cos \varphi \sin \theta \hfill \\ \end{gathered} \right. $$

with the increment *dθ* of the included angle *θ* of the strand section, the axis of the strand increases by *dl*, and the increment of the axis of the strand is *dL*, as shown in Fig. 4.

Then we can get some related formulas as follows:

7 $$ dl = \frac{dL}{{\cos \alpha }} $$

8 $$ dL = \frac{rd\varphi }{{\tan \alpha }} $$

From these equations, the following can be obtained:

9 $$ dL = \left( {\frac{D}{2} + r\cos \varphi } \right)d\theta $$

Then, according to Eqs. (7)–(9), the following equation can be given:

10 $$ d\theta = \frac{1}{{\tan \alpha \cdot \left( {\frac{D}{2r} + \cos \varphi } \right)}} \cdot d\varphi $$

So Eq. (11) can be obtained by integrating Eq. (10)

11 $$ \theta = \frac{2}{{\tan \alpha \cdot \sqrt {\frac{{D^{2} }}{{4r^{2} }} - 1} }} \cdot \arctan \frac{{\left( {\frac{D}{2r} - 1} \right) \cdot \tan \left( {\frac{\varphi }{2}} \right)}}{{\sqrt {\frac{{D^{2} }}{{4r^{2} }} - 1} }} $$

Combining Eqs. (6) and (11), we can obtain the first and second derivatives of the curvilinear parameter equation, which can be considered as the analytic equation for curvilinear motion of a point in space. Then, the first and the second derivatives of the curvilinear parameter equation are velocity and acceleration respectively. At the same time, Eq. (14) can be given from Eqs. (12) and (13) simultaneously.

12 $$ \frac{1}{\rho } = \frac{{a_{r} }}{{v^{2} }} $$

13 $$ a_{r} = \sqrt {\vec{a}^{2} - \left( {\vec{a} \cdot \frac{{\vec{v}}}{{\left| {\vec{v}} \right|}}} \right)^{2} } $$

14 $$ \frac{1}{\rho } = \sqrt {\frac{{\left( {x^{\prime 2} + y^{\prime 2} + z^{\prime 2} } \right) \cdot \left( {x^{\prime \prime 2} + y^{\prime \prime 2} + z^{\prime \prime 3} } \right) - \left( {x^{\prime } \cdot x^{\prime \prime } + y^{\prime } \cdot y^{\prime \prime } + z^{\prime } \cdot z^{\prime \prime } } \right)^{2} }}{{\left( {x^{^{\prime}2} + y^{^{\prime}2} + z^{^{\prime}2} } \right)^{3} }}} $$

Because the curvature expression is too complicated, it is appropriate to use numerical method to solve its corresponding value. The curvature at a certain point of any strand in a strand with a fixed radius of curvature can be obtained from Eq. (14), and the stress at any position on this section of the strand can be obtained according to Eq. (5).

### Construction of stress analysis model between strands of conductor

#### General assumption of conductor model

Due to the special structure of ACSR, the internal stress of conductor is very complicated when it is suppressed to external load. As a result, when establishing the conductor model, we need to make necessary assumptions to get a relatively simplified conductor model. In this paper, the following assumptions are proposed for the conductor model^29–31^.

1. Symmetry hypothesis
  The structure of ACSR determines the symmetry between strands and the similarity between adjacent layers. Therefore, it can be used to study a single strand so as to extend it to the same layer of strand, and then to study the adjacent layer of strand, so as to extend it to all layers of strand.
2. End hypothesis
  For the study of a section of conductor, it can be considered that one end of the conductor is fixed, and the other end only has axial degrees of freedom. This assumption is mostly applied in line with the actual situation.
3. Conductor contact hypothesis
  According to the geometric structure of ACSR, it can be considered that the contact type between the central strand and the adjacent strand is line contact, the contact type between the same strands is line contact, and the contact type between the adjacent strands is point contact.
4. Helix angle hypothesis
  Under the action of load, the ACSR will have relatively small torsion, but the change of torsion angle is very small, so the small change of spiral angle can be ignored.
5. Cross section assumption

In the process of tension setting out, the conductor will stretch or bend because of the complex external load, but the radial dimension of the conductor changes relatively little, so it can be assumed that the cross-sectional dimension of ACSR does not change.

#### The basic idea of constructing conductor model

Based on the theoretical basis of this chapter, the influence of envelope angle, tension load and friction on the stress characteristics of aluminum strand can be discussed. Therefore, in order to conduct more in-depth research, it is necessary to establish a reasonably simplified conductor model and consider the following aspects:

1. Simplified treatment of inner steel core
  Most of the damage of the conductor is the damage of the aluminum strand, so the stress characteristics of the aluminum strand should be considered in the study of the stress characteristics of the conductor. Based on this simplification, the inner steel core of the conductor can be simplified as a rigid cylinder, and then the inter strand stress characteristics of each layer of aluminum strand of the conductor can be studied.
2. Consideration of building model step by step
  When the whole conductor model is used to study the stress characteristics of the conductor, it is not clear to analyze the stress characteristics of each layer of the aluminum strand. Therefore, it can be considered to construct the conductor model in two steps, that is, to establish the same layer of the strand model and the adjacent layer of the strand model for research. Then, the stress characteristics of the aluminum strand part of the whole conductor can be obtained by the way of induction and recursion.
3. Simplified treatment of the same layer strand model
  The conductor has the symmetry of structure. When building the same layer strand model, the inner steel core can be simplified as a rigid cylinder, and three adjacent strand models of single layer can be built for researching. This model can directly indicate the stress state of the same ply in bending state.
4. Simplified treatment of adjacent layer strands model
  Considering the contact, friction, extrusion and other factors between the layers of aluminum strands, the inner steel core can be simplified as a rigid cylinder, and then the inner aluminum strands can be built outside the steel core. At the same time, the single-layer three adjacent aluminum strands model can be built at the outermost layer for research. This model can better reflect that in the bending state, due to the extrusion of the inner conductor Distribution characteristics of stress state caused by contact.
5. Research process of stress characteristics of aluminum strand

After the conductor model is established, the stress characteristics of aluminum strand are investigated through numerical simulation analysis. Finally, the mechanical test and stress simulation analysis of the conductor passing the pulley can be carried out based on the test platform of the research group.

#### Conductor model building process

**Figure 5:**
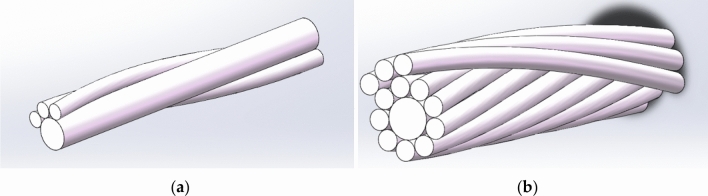
Step-by-step simplified diagram of conductor model (**a**) The same layer strand model; (**b**) Adjacent layer strands model.

In order to study the stress characteristics of aluminum strand under different envelope angle, different tension load and different friction conditions when ACSR passes through the pulley. Taking the JL/G2A-400–45/7 ACSR as the research object and using the helix command in SolidWorks, we can establish the model of the same layer of strand and the model of the adjacent layer of strand for relevant research and analysis. The length of the conductor is taken as 200 mm, and the conductor model is shown in Fig. 5.

**Figure 6:**
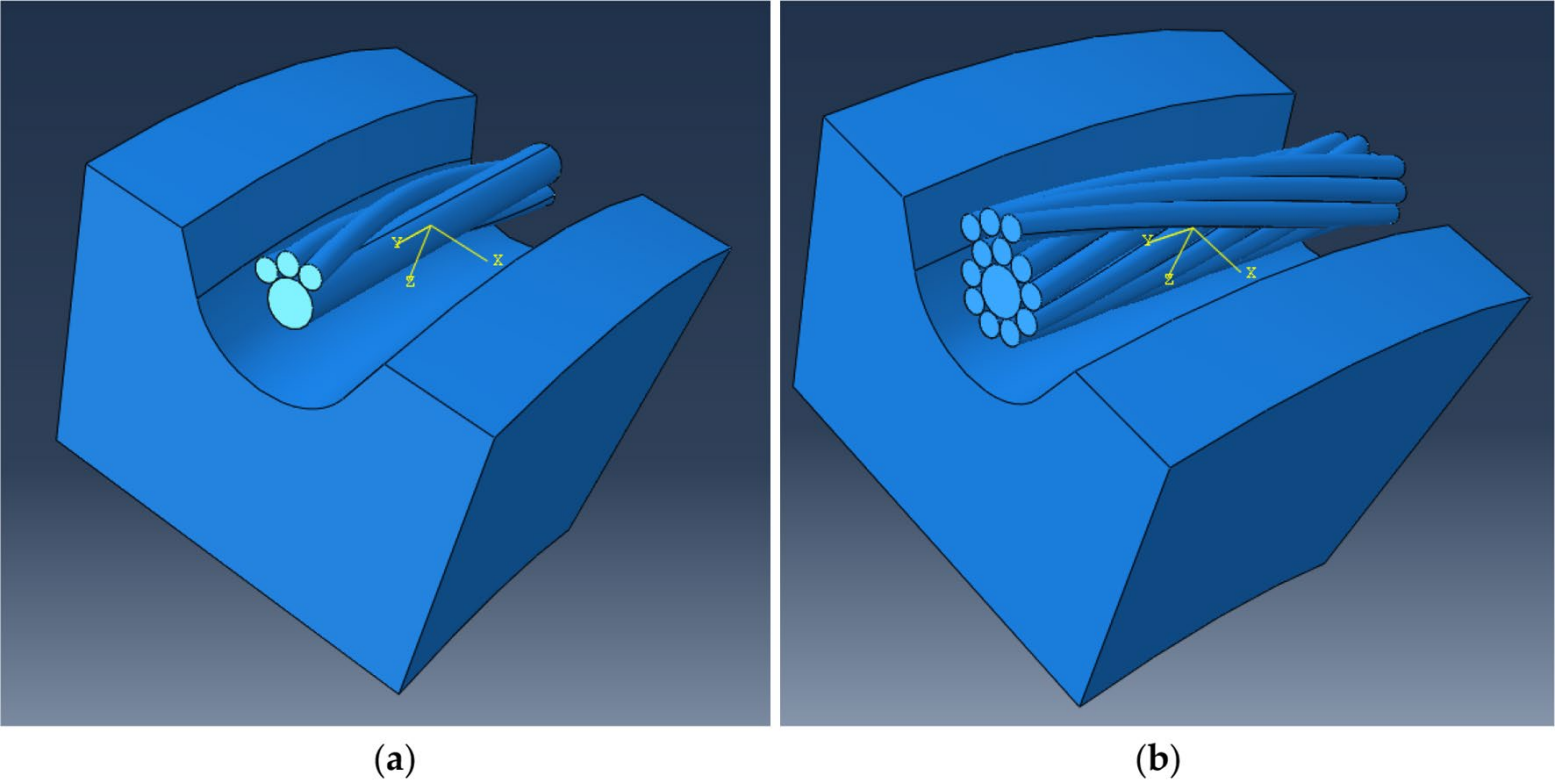
Finite element model of the conductor passing through pulley (**a**) The model of the same layer strand passing through pulley; (**b**) The model of adjacent layer strands passing through pulley.

After the construction of the conductor model, a suitable pulley model is established. The pulley adopts single R-groove type, and the groove bottom diameter is D_C_ = 560 mm. It is then imported into ABAQUS for simulation analysis (as shown in Fig. 6). It can be seen that there are many interactions among the components of the model, so when setting the correlation, the dynamic method can be used to automatically generate the interaction between components. The tangent behavior of contact attribute is set as penalty function, and the normal behavior is selected by default. When the model is solved, c3d8r mesh is used to constrain all the degrees of freedom of one end of the conductor, and the other end only retains the degrees of freedom in the axial direction, and then the displacement load is applied to the conductor by the way of pulley extrusion.

## Study on stress characteristics of ACSR in bending state

In order to study the stress characteristics of aluminum strand under different envelope angle, different tension load and different friction conditions when ACSR passes through the pulley. Taking JL/G2A-400–45/7 ACSR as the research object, the same layer strand model and the adjacent layer strands model are established for relevant research and analysis.

### Parameters of conductor structure and design of research scheme

#### Structural parameters of conductor

The JL/G2A-400–45/7 ACSR is composed of 7 steel strands and 45 aluminum strands, which are twisted in 5 layers, with an outer diameter of 26.88 mm. The relevant parameters of each layer of conductor are shown in Table 1.

**Table 1:** Structural parameters of JL/G2A-400–45/7 ACSR.

Layer/Number	Material	Diameter of single strand/mm	External diameter/mm	Pitch diameter ratio	Pitch distance/mm	Poisson’s Ratio	Elastic modulus/GPa
First lever/1	steel	2.24	2.24	–	–	0.28	196
Second level/6	steel	2.24	6.72	21	141.12	0.28	196
Third lever/9	aluminum	3.36	13.44	13	174.72	0.31	59
Fourth lever/15	aluminum	3.36	20.16	12	241.92	0.31	59
Fifth lever/21	aluminum	3.36	26.88	11	295.68	0.31	59

## Research scheme design

**Figure 7:**
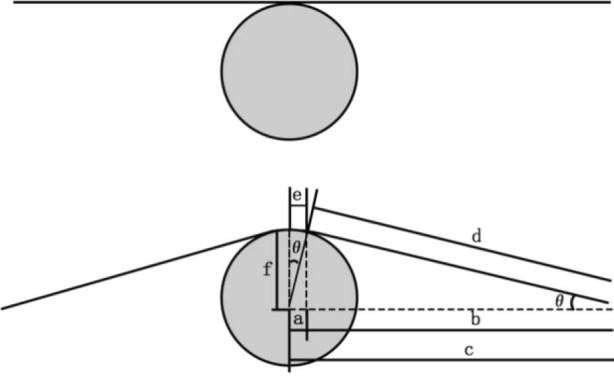
Analysis of the process of the conductor passing the pulley.

When the conductor passes through the pulley, it will be bent and deformed, and the corresponding bending stress will be generated in the aluminum strand. In order to simplify the process of the conductor passing the pulley and facilitate the numerical simulation analysis, the conductor model can be established first, and then the stress characteristics of the aluminum strand in the bending state can be studied by gradually moving the pulley to extrude the conductor. The simplified analysis of the specific traverse pulley model is shown in Fig. 7.

In Fig. 7, the specific calculation method of each parameter is as follows (unit: mm):

15 $$ a = D_{C} /2*\sin \theta $$

16 $$ b = c - a $$

17 $$ d = b/\cos \theta $$

18 $$ e = D_{C} /2*\theta /180*\pi $$

19 $$ f = b\sin \theta + D_{C} /2*\left( {1 - \cos \theta } \right) $$

where *a* describes half of the projection of the contact section of the pulley and the conductor in the horizontal direction; *b* expresses the horizontal projection from the tangent position of the pulley and the conductor to one end of the conductor; *c* denotes the horizontal distance from the center of the pulley to one end of the conductor; *d* is the distance from the tangent position of the pulley and the conductor to one end of the conductor; *e* is half of the arc length corresponding to the envelope angle; The moving distance of the pulley is defined by *f*; *θ* is half of the envelope angle; *D*_*C*_ is the groove bottom of the pulley Diameter.

### Simulation analysis of the stress state of the same layer strand

Because the stress of the aluminum strand is mainly affected by the envelope angle between the conductor and the pulley, the tension load and the friction between the strands when the conductor passes through the pulley, the influence of these three aspects on the stress of the aluminum strand should be studied in the simulation analysis of the stress state of the same layer strand.

#### The influence of envelope angle on the tress characteristics of aluminum strand

**Figure 8:**
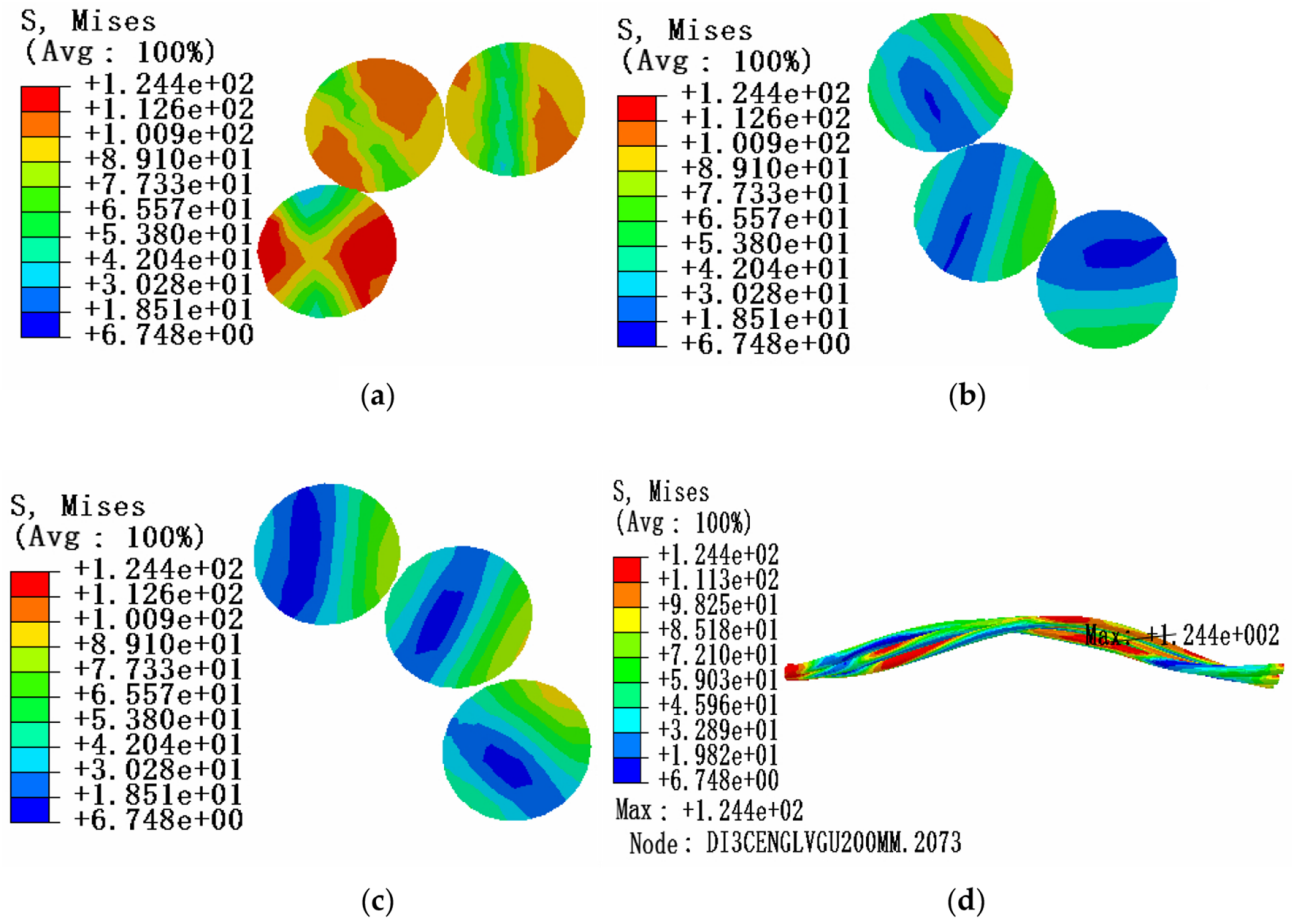
Equivalent stress cloud diagram of aluminum strand at 20% RTS (**a)** Equivalent stress cloud diagram at maximum stress; (**b**) Equivalent stress cloud diagram at 1 times the pitch diameter; (**c**) Equivalent stress cloud diagram at 0.5 times the pitch diameter; (**d**) Overall equivalent stress cloud diagram of aluminum strand.

Due to the influence of local stress caused by end face fixed constraint, in order to study the stress characteristics of aluminum strand, three sections of the same layer strand model are selected for stress analysis: the position of maximum stress, the position of 0.5 times pitch diameter and the position of 1 times pitch diameter. The friction coefficient is 0.1. The tension load is 20% of the rated tensile strength, the envelope angle of conductor and pulley is 15°, and the equivalent stress (von-Mises stress) cloud diagram is shown in Fig. 8.

The envelope angle between the conductor and the pulley is changed for analysis and comparison, and the results are shown in Table 2.

**Table 2:** Maximum equivalent stress of aluminum strand under different envelope angles.

Tension load	Friction coefficient	Envelope angle/°	Maximum stress value/MPa
20%RTS	0.1	5	108.92
20%RTS	0.1	10	119.86
20%RTS	0.1	15	124.39
20%RTS	0.1	20	127.02
20%RTS	0.1	25	132.19

According to the comparative analysis of the above simulation results, it can be seen that under the tension load of 20% RTS, the equivalent stress in the cross section of aluminum strand decreases from the edge position to the neutral layer, and the maximum equivalent stress is located at the contact position of aluminum strand and steel core. Therefore, there is a high possibility of aluminum strand damage and the larger the envelope angle between the conductor and the pulley, the greater the internal stress of aluminum strand.

#### The influence of tension load on the stress characteristics of aluminum strand

**Figure 9:**
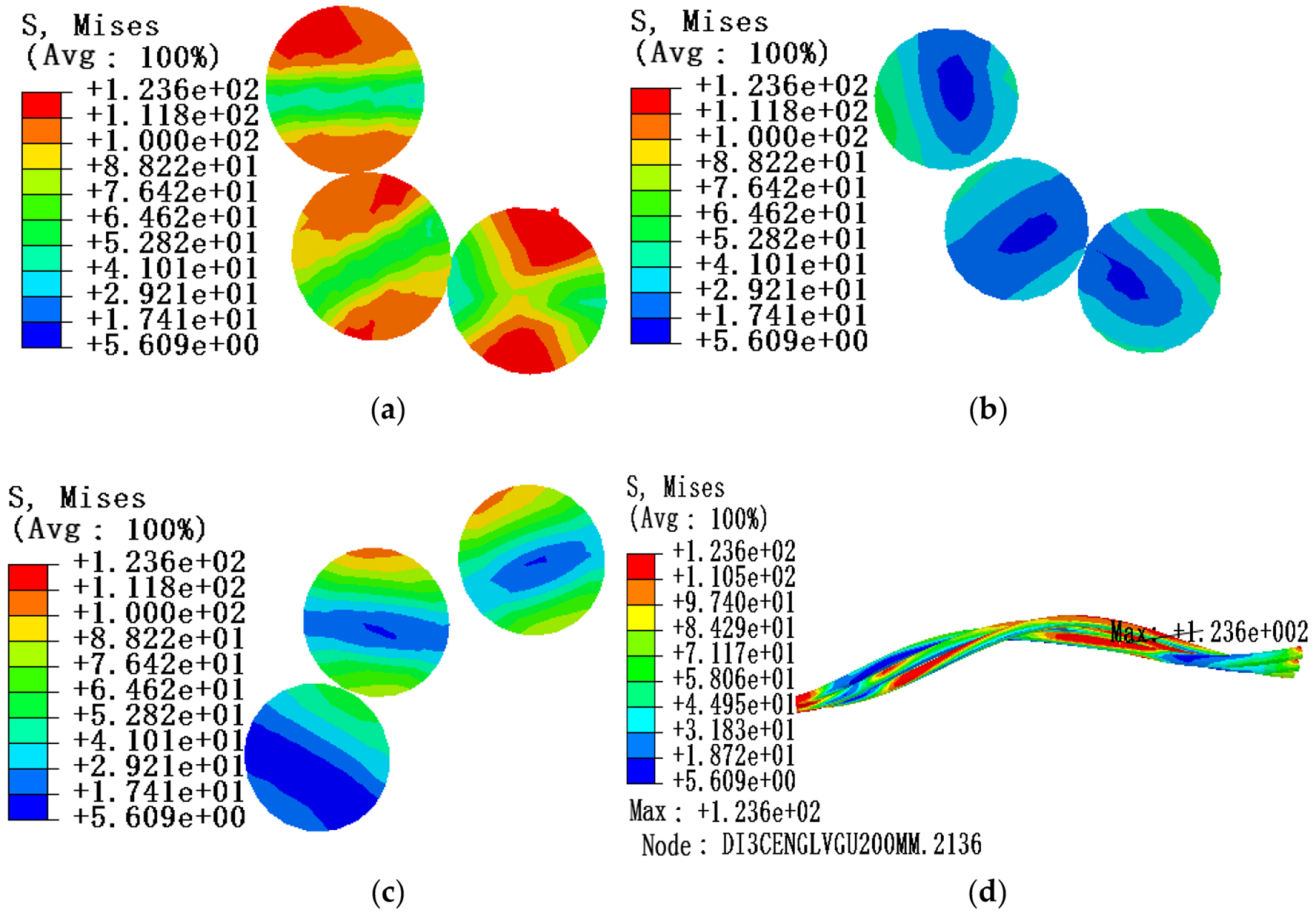
Equivalent stress cloud diagram of aluminum strand at 10% RTS (**a)** Equivalent stress cloud diagram at maximum stress; (**b**) Equivalent stress cloud diagram at 1 times the pitch diameter; (**c**) Equivalent stress cloud diagram at 0.5 times the pitch diameter; (**d**) Overall equivalent stress cloud diagram of aluminum strand.

Under the condition of a certain envelope angle, the influence of tension load on the stress characteristics of aluminum strands in bending state can be studied. In the same way, three sections of conductor model are selected for stress analysis: the position of maximum stress, the position of 0.5 times of pitch diameter and the position of 1 times of pitch diameter. The friction coefficient is 0.1. The tension load is 10% of the rated tensile strength, the envelope angle of conductor and pulley is 15°, and the equivalent stress cloud diagram is shown in Fig. 9.

The tension load is changed for analysis and comparison, and the results are shown in Table 3.

**Table 3:** Maximum equivalent stress of aluminum strand under different tension loads.

Tension load	Friction coefficient	Envelope angle/°	Maximum stress value/MPa
10%RTS	0.1	15	123.63
20%RTS	0.1	15	124.39
30%RTS	0.1	15	125.58

According to the comparison and analysis of the above simulation results, under the condition of 15° envelope angle between the conductor and the pulley, the stress value in the cross section of aluminum strand decreases from the edge position to the neutral layer, and the larger the tension load on the aluminum strand, the greater the internal stress on the aluminum strand, but the influence of the tension load on the aluminum strand is relatively small compared with the envelope angle.

#### The influence of friction on the stress characteristics of aluminum strand

**Figure 10:**
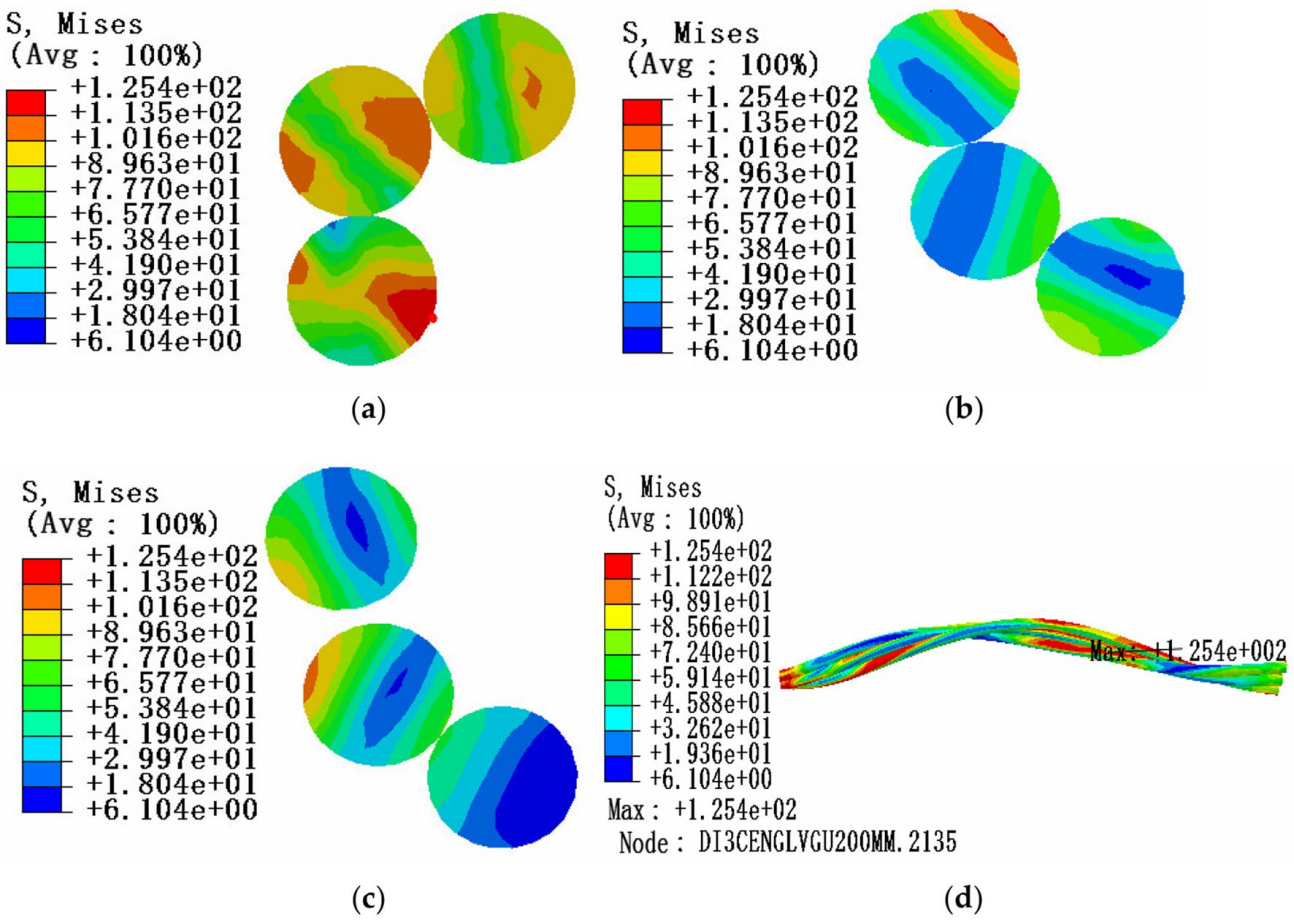
Equivalent stress cloud diagram of aluminum strand without friction (**a)** Equivalent stress cloud diagram at maximum stress; (**b**) Equivalent stress cloud diagram at 1 times the pitch diameter; (**c**) Equivalent stress cloud diagram at 0.5 times the pitch diameter; (**d**) Overall equivalent stress cloud diagram of aluminum strand.

When the envelope angle and tension load of the conductor and pulley are fixed, the influence of friction on the stress characteristics of the aluminum strand in bending state can be studied. Three sections of the conductor model are selected for stress analysis: the position of the maximum stress, the position of 0.5 times the pitch diameter and the position of 1 times the pitch diameter. At this time, considering the situation of no friction. The tension load is 20% of the rated tensile strength, the envelope angle of conductor and pulley is 15°, and the equivalent stress cloud diagram is shown in Fig. 10.

From Fig. 8, it shows the equivalent stress cloud diagram of aluminum strand with friction. Then comparing the equivalent stress cloud diagram of aluminum strand with friction to the case without friction, the results are shown in Table 4.

**Table 4:** Maximum equivalent stress of aluminum strand with or without friction.

Tension load	Friction coefficient	Envelope angle/°	Maximum stress value/MPa
20%RTS	0	15	125.43
20%RTS	0.1	15	124.39

According to the comparison and analysis of the above simulation results, it can be known that when the conductor is subjected to a tension load of 20% RTS and when the friction is considered, the stress value of the aluminum strand is smaller compared to the case without considering the friction. At the same time, it is shown that the friction between the aluminum strands can weaken the equivalent stress in the cross section to a certain extent.

### Simulation analysis of the stress state of adjacent layer strands

#### The influence of envelope angle on the tress characteristics of aluminum strand

**Figure 11:**
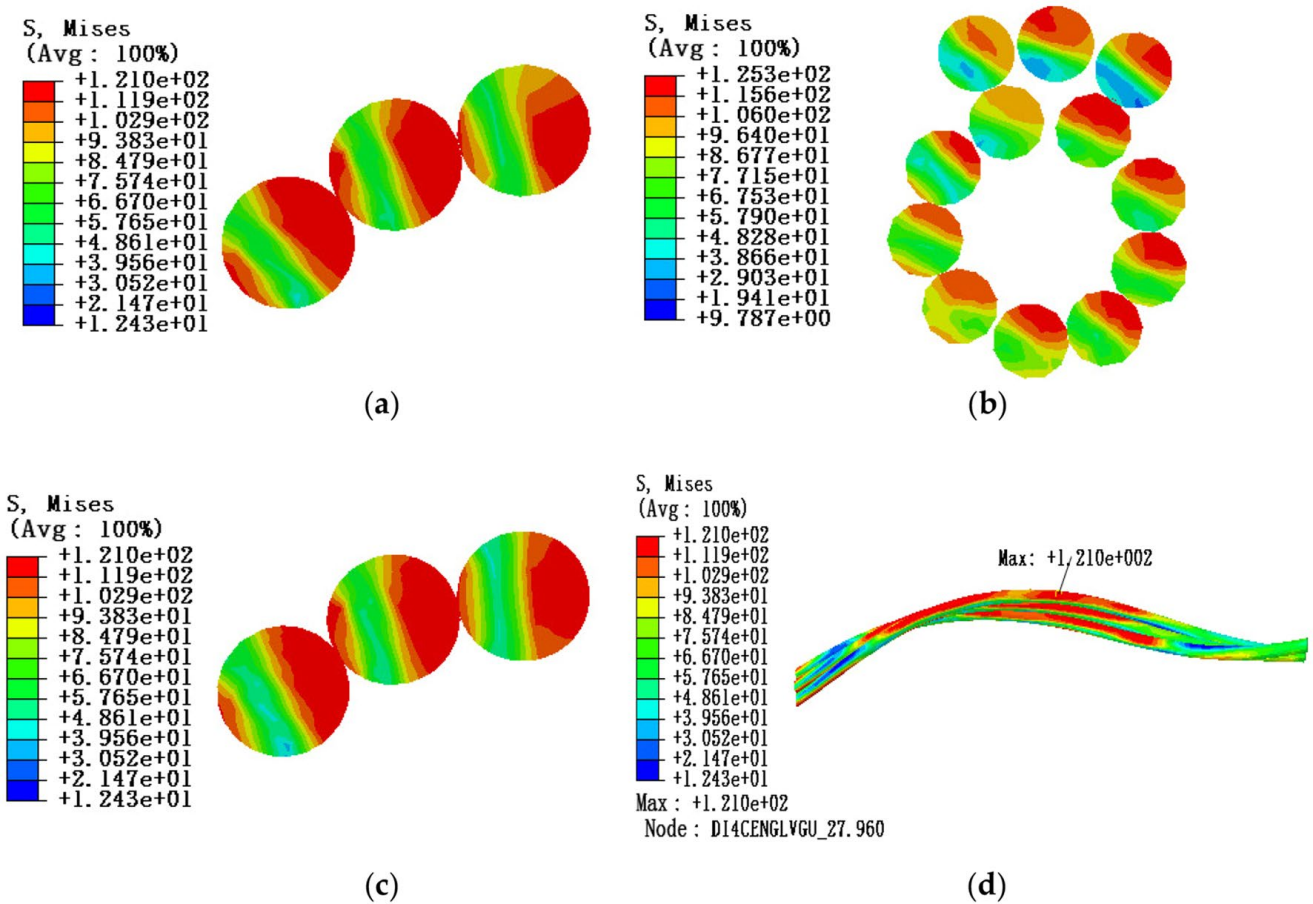
Equivalent stress cloud diagram of aluminum strand at 20% RTS (**a)** Equivalent stress cloud diagram at maximum stress; (**b**) Equivalent stress cloud diagram at the middle point section; (**c**) Equivalent stress cloud diagram at 0.5 times the pitch diameter; (**d**) Overall equivalent stress cloud diagram of aluminum strand.

Under the influence of local stress caused by end face fixed constraint, in order to study the stress characteristics of aluminum strand, three sections of the adjacent layer strands model are selected for stress analysis: the position of maximum stress, the middle point and the 0.5 times pitch diameter. The friction coefficient is 0.1. The tension load is 20% of the rated tensile strength, the envelope angle of conductor and pulley is 15°, and the equivalent stress cloud diagram is shown in Fig. 11.

The envelope angle between the conductor and the pulley is changed for analysis and comparison, and the results are shown in Table 5.

**Table 5:** Maximum equivalent stress of aluminum strand under different envelope angles.

Tension load	Friction coefficient	Envelope angle/°	Maximum stress value/MPa
20%RTS	0.1	5	104.83
20%RTS	0.1	10	113.26
20%RTS	0.1	15	120.97
20%RTS	0.1	20	123.46
20%RTS	0.1	25	128.71

According to the comparative analysis of the above simulation results, it can be known that under the tension load of 20% RTS, the stress value in the cross section of aluminum strand decreases from the edge position to the neutral layer, and the maximum equivalent stress appears in the adjacent aluminum strand at the contact position and comparing the stress of the same layer of strands, it can be seen that the equivalent stress of the aluminum strands increases from the outer layer to the inner layer, that is, the equivalent stress value of the outer layer of aluminum strands is smaller, and the equivalent stress value of the inner layer is larger. Therefore, the possibility of damage to the aluminum strands at the contact position of the adjacent aluminum strands is greater, and the inner aluminum strands are more easily damaged than the outer aluminum strands. In addition, from Table 5, it can be seen that the larger the envelope angle between the conductor and the pulley, the greater the equivalent stress of the aluminum strand.

#### The influence of tension load on the stress characteristics of aluminum strand

**Figure 12:**
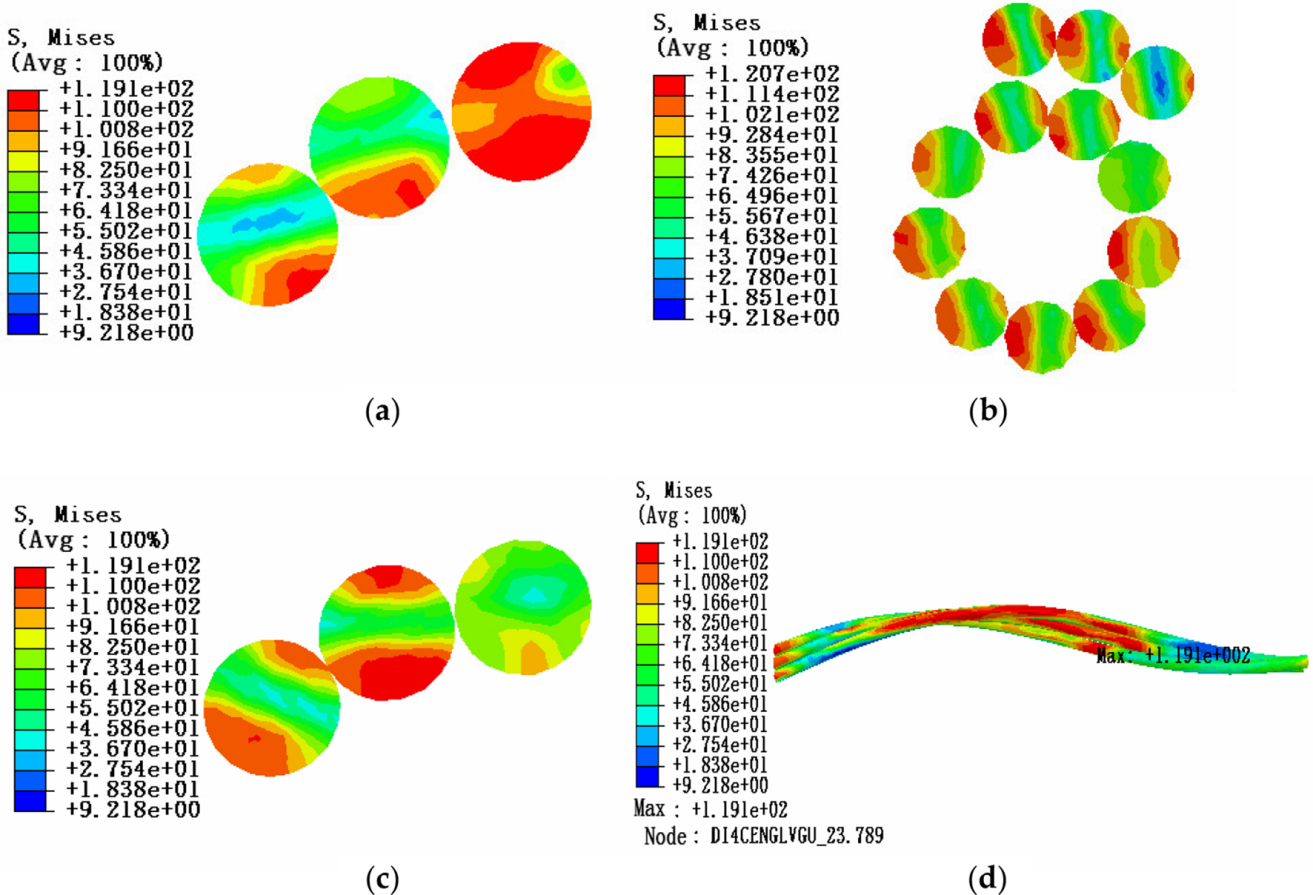
Equivalent stress cloud diagram of aluminum strand at 10% RTS (**a)** Equivalent stress cloud diagram at maximum stress; (**b**) Equivalent stress cloud diagram at the middle point section; (**c**) Equivalent stress cloud diagram at 0.5 times the pitch diameter; (**d**) Overall equivalent stress cloud diagram of aluminum strand.

Under the condition of a certain envelope angle, the influence of tension load on the stress characteristics of aluminum strand in bending state can be studied. In the same way, three sections of the adjacent layer strands model are selected for stress analysis: the position of maximum stress, the middle point and the 0.5 times pitch diameter. The friction coefficient is 0.1. The tension load is 10% of the rated tensile strength, the envelope angle of the conductor and the pulley is 15°, and the equivalent stress cloud diagram is shown in Fig. 12.

The tension load is changed for analysis and comparison, and the results are shown in Table 6.

**Table 6:** Maximum equivalent stress of aluminum strand under different tension loads.

Tension load	Friction coefficient	Envelope angle/°	Maximum stress value/MPa
10%RTS	0.1	15	119.14
20%RTS	0.1	15	120.97
30%RTS	0.1	15	123.08

According to the comparison and analysis of the above simulation results, it can be known that under the 15° envelope angle between the conductor and the pulley, the equivalent stress in the cross section of aluminum strand decreases from the edge position to the neutral layer, and the maximum equivalent stress appears in the adjacent aluminum strand at the contact position, and comparing the stress of the same layer of strands, it can be seen that the equivalent stress of the aluminum strands tends to increase from the outer layer to the inner layer. In addition, the larger the tension load on the aluminum strand, the greater the equivalent stress of the aluminum strand, but the effect of the tension load on the aluminum strand is relatively small compared to the envelope angle.

#### The influence of friction on the stress characteristics of aluminum strand

**Figure 13:**
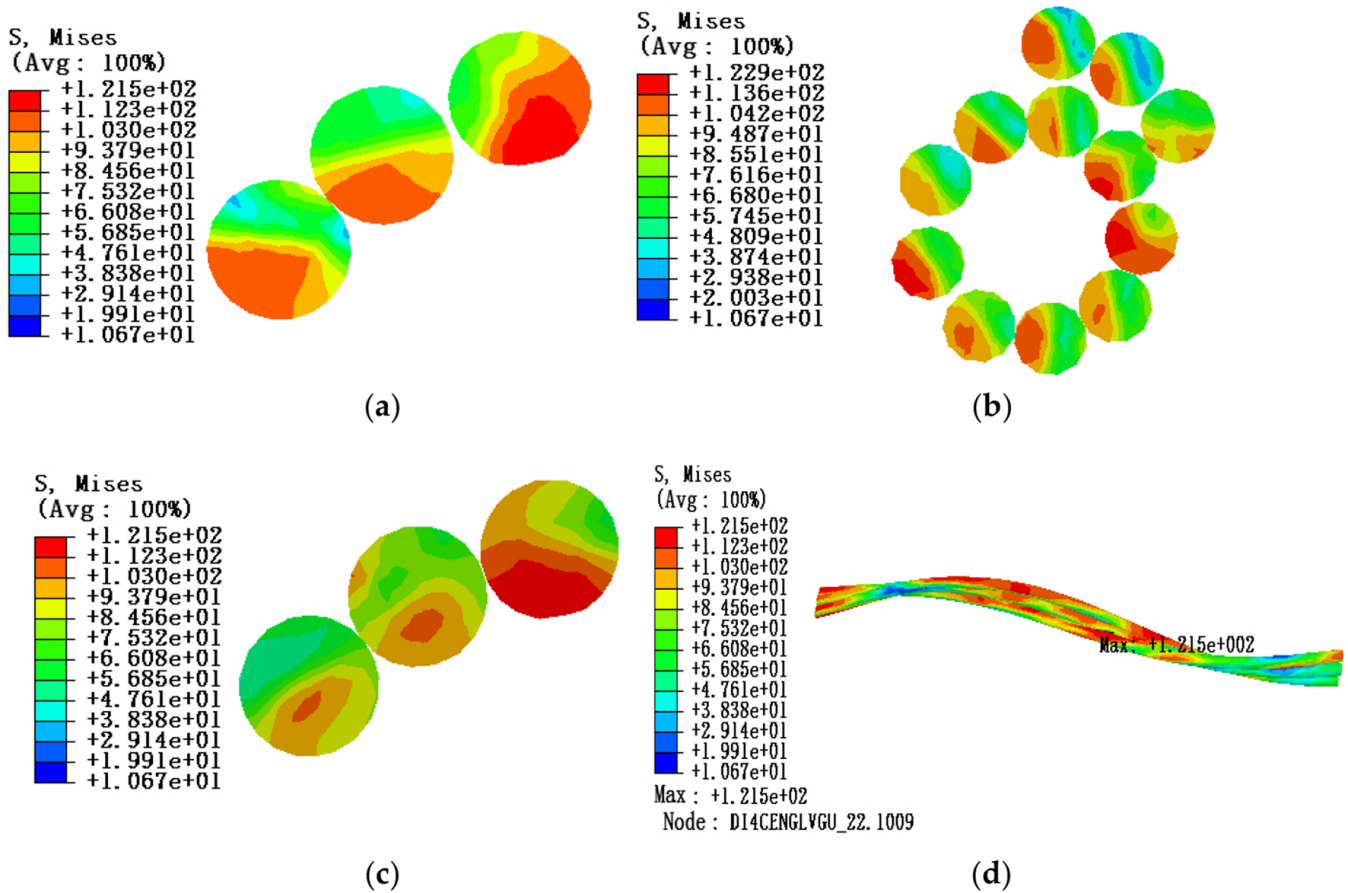
Equivalent stress cloud diagram of aluminum strand without friction (**a)** Equivalent stress cloud diagram at maximum stress; (**b**) Equivalent stress cloud diagram at the middle point section; (**c**) Equivalent stress cloud diagram at 0.5 times the pitch diameter; (**d**) Overall equivalent stress cloud diagram of aluminum strand.

Under the condition that the envelope angle of the conductor and the pulley and the tension load are constant, the influence of friction on the stress characteristics of the aluminum strand in bending state can be studied. Three sections of the adjacent layer strands model are selected for stress analysis: the position of the maximum stress, the position of 0.5 times the pitch diameter and the position of 1 times the pitch diameter. In this time, considering the situation of no friction, the tension load is 20% of the rated tensile strength, the envelope angle of conductor and pulley is 15°, and the equivalent stress cloud diagram is shown in Fig. 13.

From Fig. 11, it shows the equivalent stress cloud diagram of aluminum strand with friction. Then comparing the equivalent stress cloud diagram of aluminum strand with friction to the case without friction, the results are shown in Table 7.

**Table 7:** Maximum equivalent stress of aluminum strand with or without friction.

Tension load	Friction coefficient	Envelope angle/°	Maximum stress value/MPa
20%RTS	0	15	121.50
20%RTS	0.1	15	120.97

According to the comparative analysis of the above simulation results, it can be known that under the condition that the conductor is subjected to a tension load of 20% and when the friction is considered, the stress value of the aluminum strand is smaller compared to the case without considering the friction. At the same time, it shows that the friction between the aluminum strands has a certain effect on reducing the equivalent stress in the cross section. Moreover, the equivalent stress in the cross section of aluminum strand decreases from the edge position to the neutral layer and the maximum equivalent stress appeared at the contact position of adjacent aluminum strands. Comparing the stress value of the same layer of strands, it can be seen that the equivalent stress value of aluminum strands tends to increase from the outer layer to the inner layer.

## Stress characteristic test and simulation analysis of conductor passing through pulley

In order to further study the inter strand stress state of the conductor passing pulley, the JL/G1A-630–45/7 ACSR will be taken as the research object. Firstly, on the basis of the test platform of the research group, the test of the conductor passing pulley will be carried out, and the stress value of the aluminum strand under different working conditions will be measured to study the stress characteristics of the aluminum strand in the process. Then, according to the conductor model proposed in this paper, the numerical simulation analysis of the conductor passing through the pulley can be carried out to get the stress situation of the aluminum strand in the corresponding position under this state. Finally, the simulation and test are compared, which can preliminarily verify the same layer strand, as well as the preliminary study of the inter strand stress characteristics of the conductor under the bending state.

### Test related preparations

#### Test platform and test method

**Figure 14:**
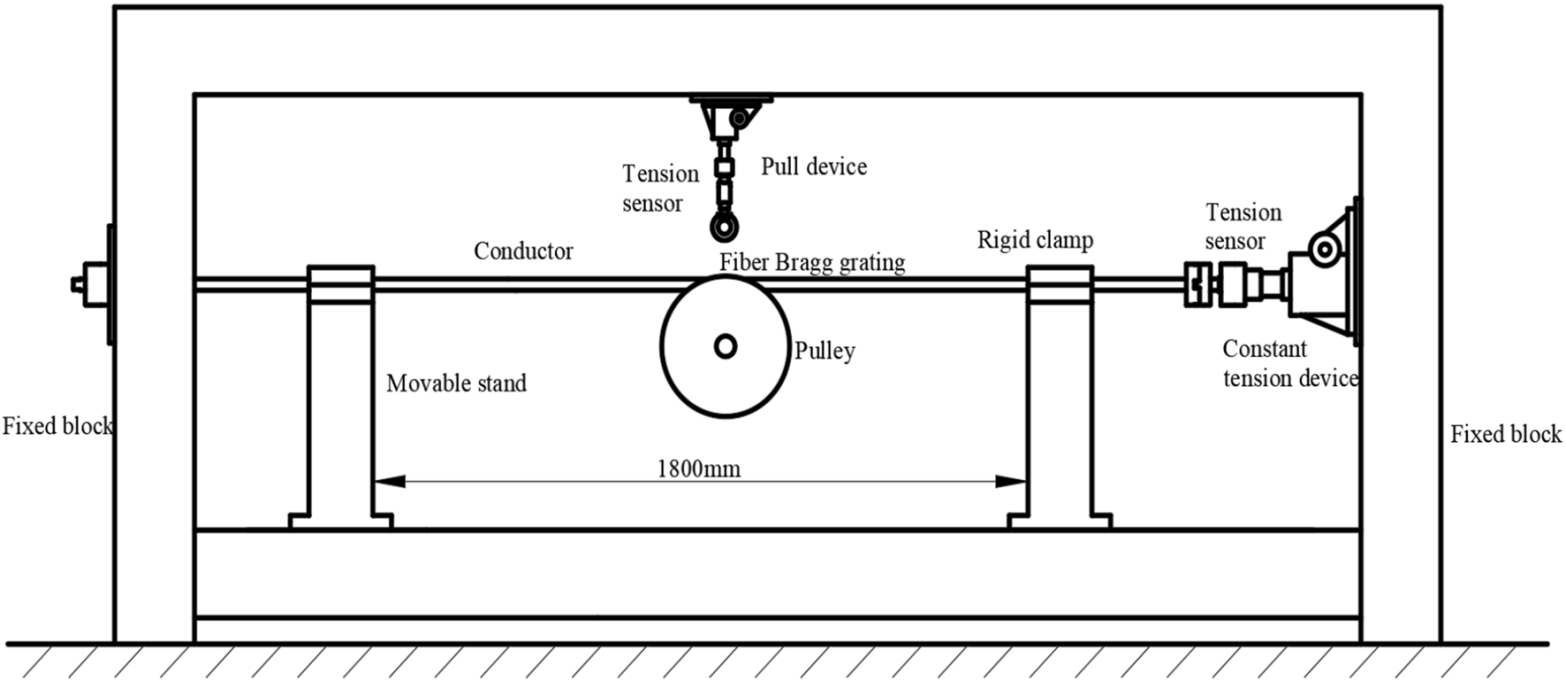
Diagram of mechanical test platform.

The test platform has a length of 4.3 m, a height of 2.3 m, and an area of about 9 m2. The schematic diagram of the test platform is shown in Fig. 14, which roughly includes four parts: fixing system, pulley system, tension load system, and signal acquisition system. The fixing system consists of fixed block, movable stand and rigid clamp, while the pulley system is composed of pulley and pull device. Then constant tension device is the main part of tension load system as well as the signal acquisition system is made up of tension sensor and fiber Bragg grating sensor.

When the conductor is continuously subjected to tension load, its internal molecular structure will produce permanent and irreversible creep lengthening. After the tension load is applied to the metal, the creep lengthening rate is relatively fast at the beginning, and then gradually slows down until it stops over time. For ACSR, after 48 h of tension load, the deformation reaches a stable state. In this test, the conductor is exerted 20% RTS by the constant tension device, and then the test was started after setting the conductor aside for two days. The test scheme is as follows:

1. At the first, ACSR should be fixed on the test platform, and then the test platform is adjusted to ensure that conductor is horizontal. Then, the wire rope is used to connect the pulley with the eyebolt. Finally, U-bolts are used to lock them tightly to prevent the pulley from falling off when the test is carried out.
2. The fiber Bragg grating sensor is pasted to the surface of the aluminum strand which can be found directly above the pulley groove. Then the signal is sent back to the demodulator so that the wavelength can be determined by computer software.
3. The tension load is imposed on the conductor by the constant tension device. In addition, the winding crank should be operated to make the pull device pull the pulley to rise vertically. Then it ought to stop operating the winding crank when the wavelength is going to change. Besides, the wavelength needs to be written down as the initial wavelength. At last, the constant tension device could be operated to satisfy the test.
4. Thorough the method of replacing the envelope angle with displacement, the displacement associated with different envelope angles can be calculated. Then turn the winding crank slowly to apply the vertical displacement to the conductor. It ought to be stopped until the envelope angle is satisfied to the test requirement, and then the wavelength needs to be taken down.
5. Repeat the test process and record the wavelength of the different envelope angles. Besides, change the tension load and repeat the test three times, after that, the final test figure is an average of the three sets of data.
6. Based on the principle of fiber Bragg grating sensor, the wavelength difference could be calculated, and then the micro-stain of the aluminum strand can be acquired through formula transformation. Therewithal, the stress value will be obtained based on the elastic modulus of aluminum.

#### Some analysis before test

**Figure 15:**
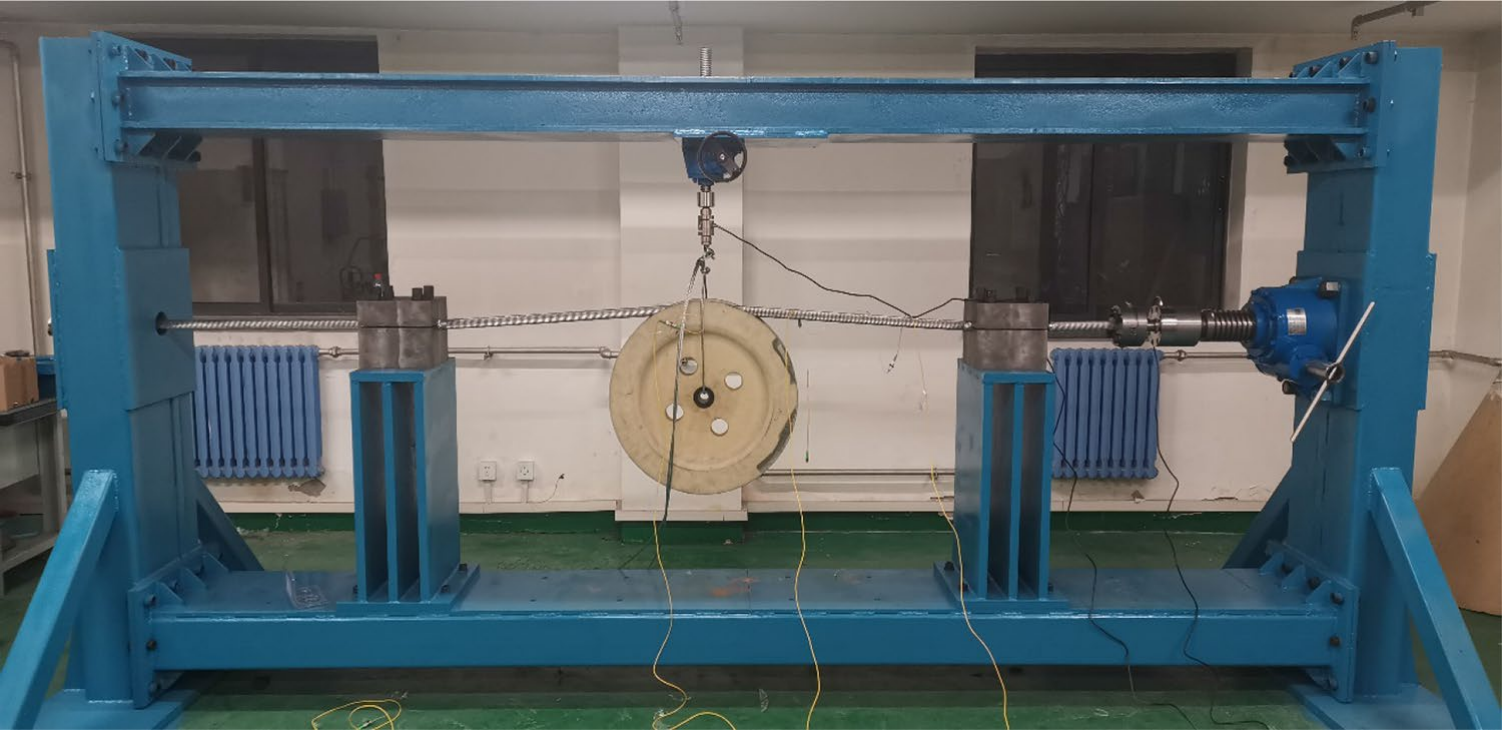
Mechanical test platform of conductor passing through pulley.

In this test, the JL/G1A-630–45/7 ACSR and single R-groove type pulley with groove bottom diameter of 550 mm are used. Both of them are the real conductor and pulley used in the actual tension setting out, so the test has high authenticity and reliability, and the test results have strong persuasion, and can preliminarily verify the accuracy of the same layer model and carry out certain research on the stress characteristics of the aluminum strand when the conductor passes by the pulley. The mechanical test platform of the conductor passing through the pulley is shown in Fig. 15.

In the process of passing the pulley, the conductor will be squeezed by the wheel groove, resulting in bending deformation. From the actual situation, the internal stress of the aluminum strand in the upper half of the wheel groove is different from that in the lower half. However, it can be seen that the curvature of the aluminum strand in the lower half of the groove is larger, while the curvature of the aluminum strand in the upper half is smaller. Similarly, according to the principle of force transmission, the extrusion pressure of the two parts of aluminum strand is basically the same, so the different local stress produced by the two parts of aluminum strand under the same extrusion pressure is generally caused by the different curvature of the two parts of aluminum strand and the different shape variables.

Based on the above analysis, in the test, only the stress of the aluminum strand in the upper half of the wheel groove needs to be tested, while the stress state of the aluminum strand in the lower half can be equivalent to the stress state of the upper half with large curvature. In addition, according to the symmetry hypothesis of the conductor structure and the same layer strand model, when testing the upper part of the aluminum strand, we can find an aluminum strand directly above to test and study, which can meet the needs of this test and satisfy the purpose of the test. In the process of data acquisition, the fiber Bragg grating acquisition system is used. By finding the wavelength difference of the corresponding test condition, the corresponding micro strain is converted, and then the internal stress value of the aluminum strand under this condition is obtained. At the same time, when arranging the measurement points of the fiber Bragg grating, the fixed constraints at both ends of the test lead will cause local stress on the aluminum strand. Therefore, the measurement point should be located at the midpoint of the aluminum strand. In this way, it is possible to avoid the test error caused by the fixed constraint at both ends of the conductor, so that the test data is more reliable. The structural parameters of JL/G1A-630–45/7 steel core aluminum strand used in this test are shown in Table 8.

**Table 8:** Structural parameters of JL/G1A-630–45/7 ACSR.

Layer/Number	Material	Diameter of single strand/mm	External diameter/mm	Pitch diameter ratio	Pitch distance/mm	Poisson’s ratio	Elastic modulus/GPa
First lever/1	Steel	2.81	2.81	-	-	0.28	190
Second level/6	Steel	2.81	8.43	20.0	168.600	0.28	190
Third lever/9	Aluminum	4.22	16.87	15.5	261.485	0.30	55
Fourth lever/15	Aluminum	4.22	25.31	13.2	334.092	0.30	55
Fifth lever/21	Aluminum	4.22	33,75	11.0	371.250	0.30	55

### Test research on conductor passing through pulley

#### Stress test of aluminum strand under different working conditions

During this test, it is obvious that the main factors that affect the internal stress state of aluminum strand are the envelope angle of conductor and pulley and the tension load of conductor during tension paying off construction. Therefore, according to the needs and purposes of the test, the influence of different envelope angle and different tension load on the stress of aluminum strand can be studied under the condition of the same conductor span (the conductor span tested here is L = 1800 mm).

Due to the limitation of test conditions and unavoidable test errors, although this test cannot be consistent with the envelope angle of the conductor in the process of passing the pulley, this test has been relatively able to satisfy the research and analysis of related problems. Therefore, when studying the influence of the envelope angle of the conductor and the pulley on the stress state of the aluminum strand, you can choose a test condition where the envelope angle of the conductor and the pulley is a = 3°, a = 6°, a = 9°, a = 12° and a = 15°. Correspondingly, when studying the influence of the conductor on the stress state of aluminum strands under different tension loads, the test conditions can be selected for the tension load T = 0kN and T = 10kN.

In determining the envelope angle of the conductor and pulley, the displacement method which has been discussed is implemented to control the envelope angle. The envelope angle is transformed into the displacement load in the vertical direction. The different envelope angle of the conductor and pulley can be obtained by applying different displacement loads to the conductor through the pulley. The specific working conditions in the test are as follows.

Test condition 1: tension load of conductor is T = 0kN.

When the tension load of ACSR in the test is T = 0kN, the wavelength of optical fiber under different envelope angle measured in the test and the converted stress value of ACSR are shown in Table 9.

**Table 9:** Stress value test of aluminum strand under tension load of 0kN.

Envelope angle/°	3°	6°	9°	12°	15°
Fiber test wavelength/nm	1533.839	1534.479	1534.646	1534.828	1534.969
Stress of aluminum strand/MPa	43.496	72.829	80.483	88.825	95.287

Test condition 2: tension load of conductor is T = 10kN.

When the tension load of ACSR in the test is T = 10kN, the wavelength of optical fiber under different envelope angle measured in the test and the converted stress value of ACSR are shown in Table 10.

**Table 10:** Stress value test of aluminum strand under tension load of 10kN.

Envelope Angle/°	3°	6°	9°	12°	15°
Fiber test wavelength/nm	1534.074	1534.626	1534.966	1535.193	1535.461
Stress of aluminum strand/MPa	54.267	79.567	95.150	105.554	117.838

#### Analysis of test results

**Figure 16:**
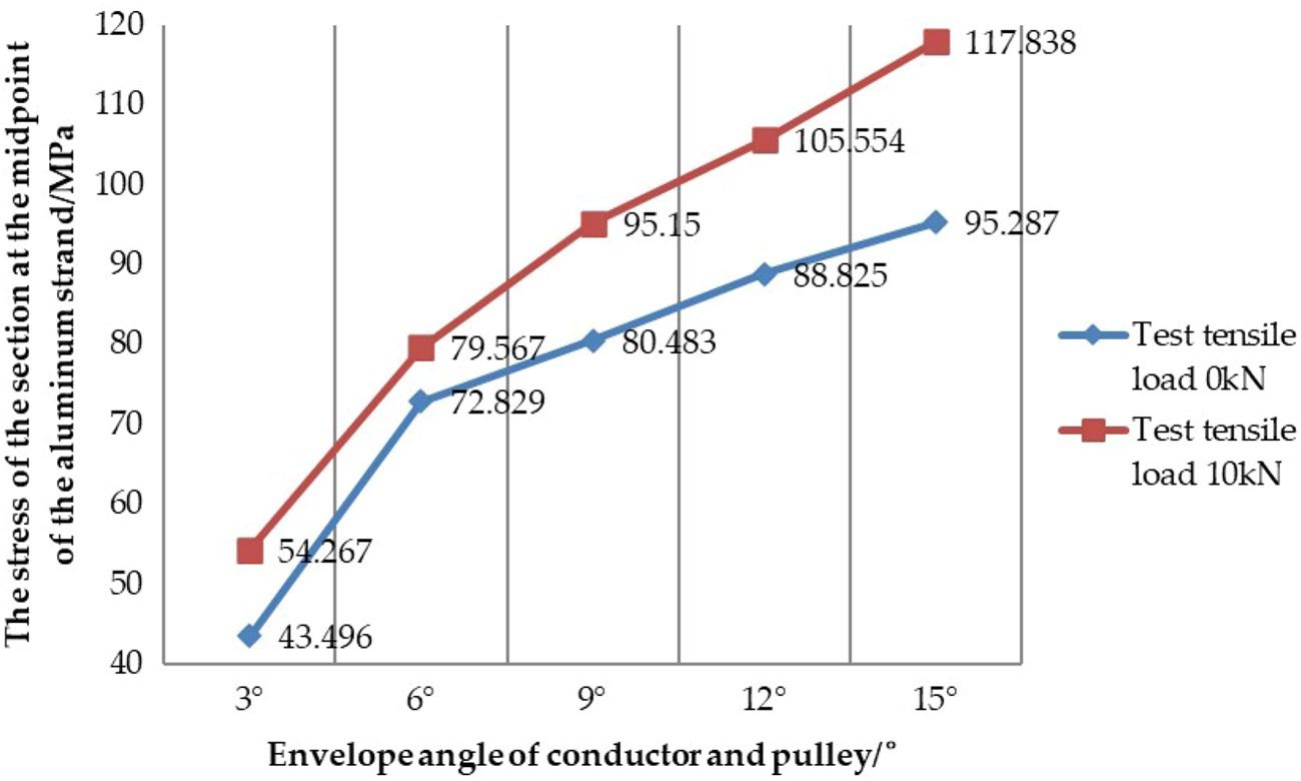
Curve diagram of the stress of the section at the midpoint of the aluminum strand with the change of the envelope angle under the test condition.

Through the test of the stress value at the midpoint of the aluminum strand under different working conditions, Tables 9 and 10 are obtained. In order to more clearly find the influence of the envelope angle and the tension load of the conductor on the internal stress of aluminum strand, the curve diagram of the stress of the section at the midpoint of aluminum strand with the change of the envelope angle can be made, when the conductor is under the test condition 1 and the test condition 2, as shown in Fig. 16.

In Fig. 16, when the tension load in the test is T = 0kN, with the increase of the envelope angle between the conductor and the pulley, the equivalent stress at the midpoint of the aluminum strand also increases positively, and the same rule appears when the tension load is T = 10kN. In addition, it can be seen from the figure that the data line chart obtained under test condition 1 is generally below the data line chart obtained under test condition 2, which shows that under the same envelope angle, the stress of the section at the midpoint of the aluminum strand line increases with the increase of the tension load, but according to the numerical comparison analysis, it is known that the envelope angle of the conductor and the pulley and the tension load of the conductor are the two main factors affecting the internal stress of the aluminum strand. And then the influence of the envelope angle on the stress value of aluminum strands is more obvious than the tension load.

### Numerical simulation analysis of conductor passing through pulley

#### Construction of simulation model

**Figure 17:**
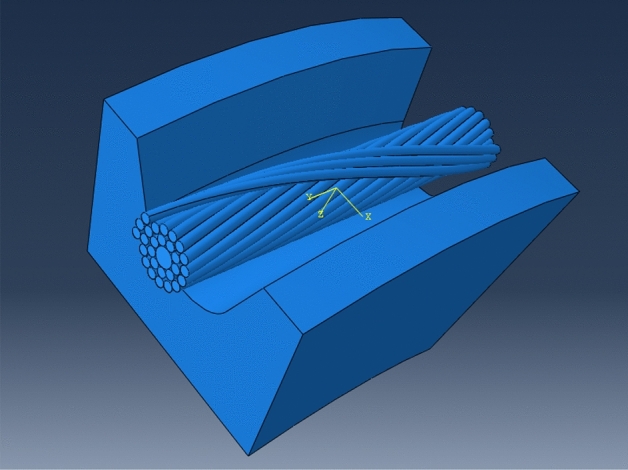
Finite element model of the conductor passing through pulley in the test.

According to the conductor model proposed in this paper, combined with the type of pulley used in this test and the relevant structural parameters of JL/G1A-630–45/7 ACSR, the model of conductor passing through the pulley consistent with the test is established. For this model, in order to compare with the test situation and save the calculation time, the length of the conductor is taken as L = 180 mm. After the model is established, it is imported into ABAQUS for numerical simulation analysis (as shown in Fig. 17). The interaction among the parts of the model is still generated automatically by the general dynamics method. In the solution, all the degrees of freedom on one end face of the conductor are constrained, and the other end face only retains the degrees of freedom in the axial direction. Then the tension load is applied according to the test needs, and finally the displacement load is applied to the conductor by the way of the pulley squeezing the conductor.

#### Simulation results of aluminum strand under different working conditions

Based on the model, two working conditions of conductor passing through the pulley in the test are simulated, and then the equivalent stress cloud diagram of the conductor passing through pulley can be obtained through the post-processing of the simulation software. In the analysis of the comparative test results and the study of the stress state of the aluminum strand under this bending state, the aluminum strand at the corresponding position in the test should be selected for research. And in the process of simulation analysis, it is necessary to carry out specific analysis corresponding to the test conditions.

Simulation condition 1: tension load of conductor is T = 0kN.

**Figure 18:**
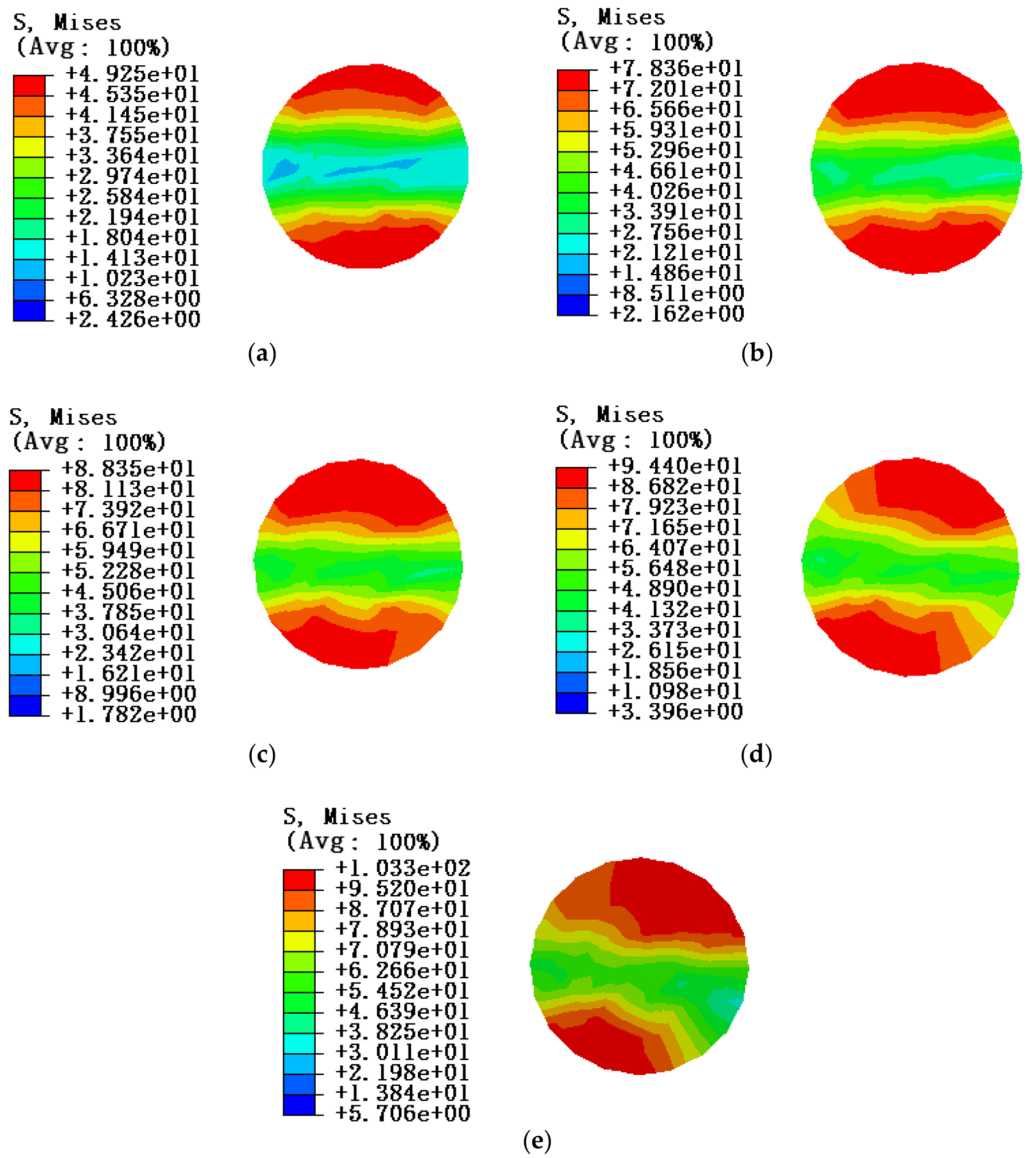
Equivalent stress cloud diagram of midpoint position of aluminum strand at tension load of 0kN (**a)** Equivalent stress cloud diagram of aluminum strand at an envelope angle of 3°; (**b**) Equivalent stress cloud diagram at an envelope angle of 6°; (**c**) Equivalent stress cloud diagram at an envelope angle of 9°; (**d**) Equivalent stress cloud diagram of aluminum strand at an envelope angle of 12°; (**e**) Equivalent stress cloud diagram of aluminum strand at an envelope angle of 15°.

Due to the influence of local stress caused by the fixed constraint of end face, when studying the stress characteristics of aluminum strand under the bending state of conductor, in order to be consistent with the test, the section at the midpoint of the aluminum strand is selected for stress analysis, and the tension load of conductor is taken as T = 0kN, and the equivalent stress cloud diagram of different envelope angles between the conductor and the pulley is shown in Fig. 18.

According to the equivalent stress cloud diagram of the numerical simulation, it can be seen that the stress distribution state of the corresponding midpoint position of the aluminum strand at different envelope angles under the condition of simulation condition 1. Therefore, in order to better analyze and compare the equivalent stress in the cross section, and so as to more intuitively explain the problem, the results of this simulation can be shown in Table 11.

**Table 11:** Stress simulation results of aluminum strand under tension load of 0kN.

Envelope Angle/°	3°	6°	9°	12°	15°
Section stress of aluminum strand/MPa	49.253	78.357	88.348	94.403	103.338

Simulation condition 2: tension load of conductor is T = 10kN.

**Figure 19:**
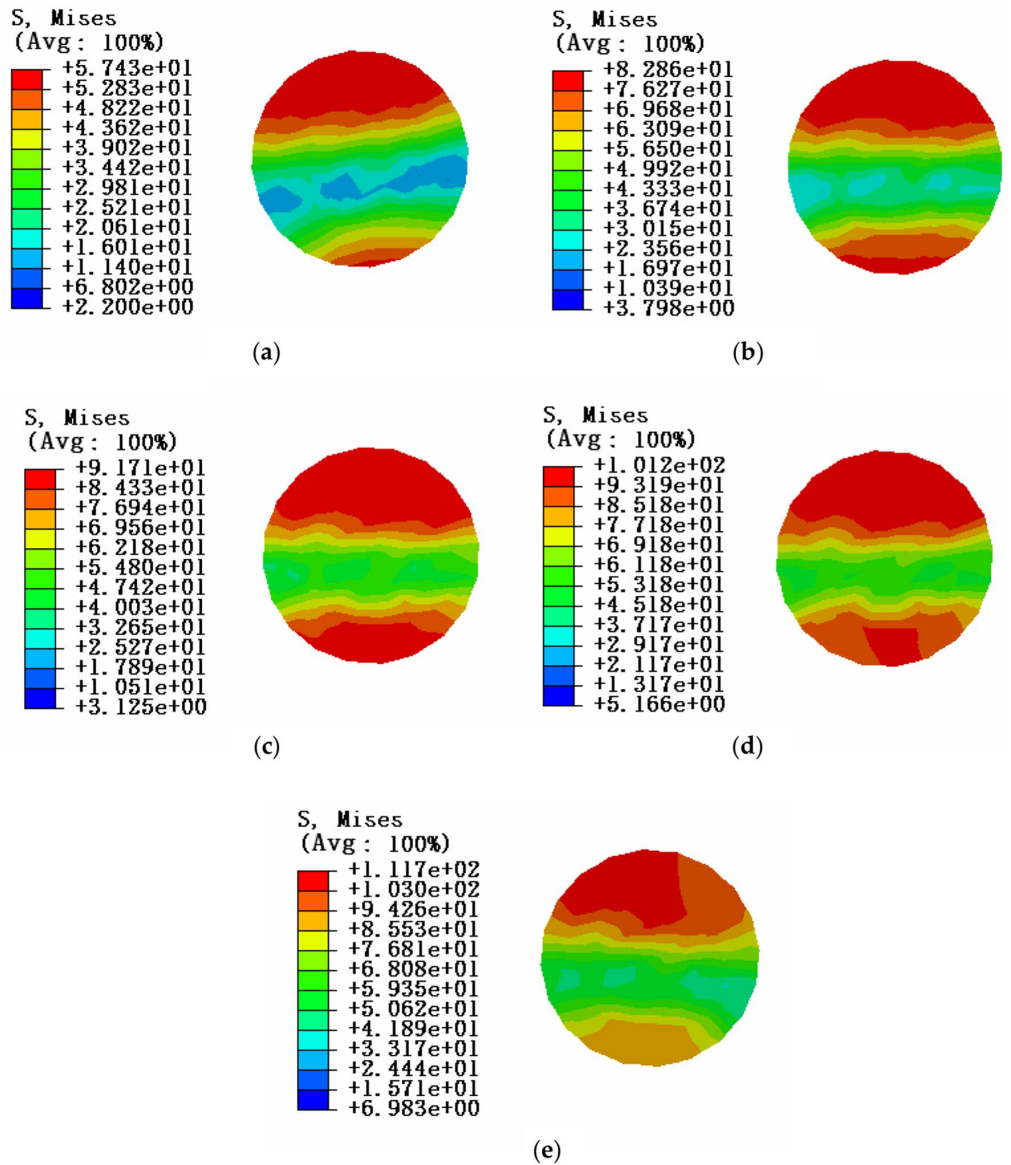
Equivalent stress cloud diagram of midpoint position of aluminum strand at tension load of 10kN (**a)** Equivalent stress cloud diagram of aluminum strand at an envelope angle of 3°; (**b**) Equivalent stress cloud diagram at an envelope angle of 6°; (**c**) Equivalent stress cloud diagram at an envelope angle of 9°; (**d**) Equivalent stress cloud diagram of aluminum strand at an envelope angle of 12°; (**e**) Equivalent stress cloud diagram of aluminum strand at an envelope angle of 15°.

Due to the influence of local stress caused by the fixed constraint of end face, when studying the stress characteristics of aluminum strand under the bending state of conductor, in order to be consistent with the test, the section at the midpoint of the aluminum strand is selected for stress analysis, and the tension load of conductor is taken as T = 0kN, and the equivalent stress cloud diagram of different envelope angles between the conductor and the pulley is shown in Fig. 19.

According to the equivalent stress cloud diagram of the numerical simulation, it can be seen that the stress distribution state of the corresponding midpoint position of the aluminum strand is at different envelope angles under the condition of simulation condition 2. As with simulation condition 1, the results of this simulation can be shown in Table 12.

**Table 12:** Stress simulation results of aluminum strand under tension load of 10kN.

Envelope Angle/°	3°	6°	9°	12°	15°
Section stress of aluminum strand/MPa	57.429	82.857	91.707	101.189	111.718

#### Analysis of simulation results

**Figure 20:**
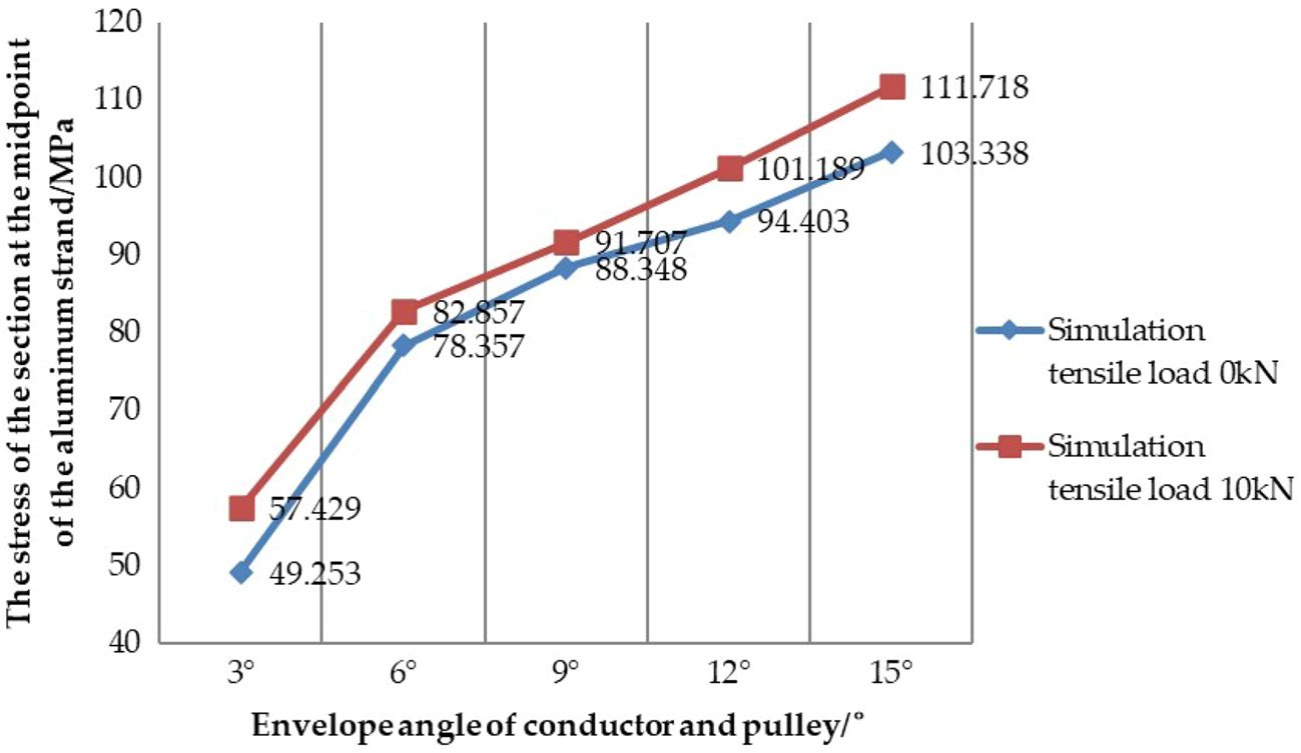
Curve diagram of the stress of the section at the midpoint of the aluminum strand with the change of the envelope angle under the simulation condition.

On the basis of the previous test, the numerical simulation of the conductor model proposed in this paper is used to simulate the stress state at the midpoint of the aluminum strand under different working conditions, and the equivalent stress cloud diagrams under different envelope angles are obtained, and the simulation results are shown in Table 11 and Table 12. In order to analyze the simulation results clearly, the same research method as the test results is used to make the curve diagram of the stress of the section at the midpoint of the aluminum strand with the change of the envelope angle when the conductor is under the simulation condition 1 and the simulation condition 2, as shown in Fig. 20.

It can be seen from the equivalent stress cloud diagram at the midpoint of the aluminum strand that the equivalent stress in the cross section of aluminum strand decreases from the outside to the center of the section, that is, the stress in the neutral layer of a single aluminum strand is smaller and the stress at the edge is larger. The law consistent with the test results is shown in Fig. 20: under the two simulation conditions, with the increase of the envelope angle between the conductor and the pulley, the equivalent stress at the midpoint of the aluminum strand increases positively; By comparing the data from simulation condition 1 and simulation condition 2, it can also be seen that with the same envelope angle, the stress of the section at the midpoint of the aluminum strand increases with the increase of the load on the axis of the conductor, and the influence of the envelope angle on the stress value of the aluminum strand is more obvious than that of the tension load.

### Comparative analysis of test results and simulation results

**Figure 21:**
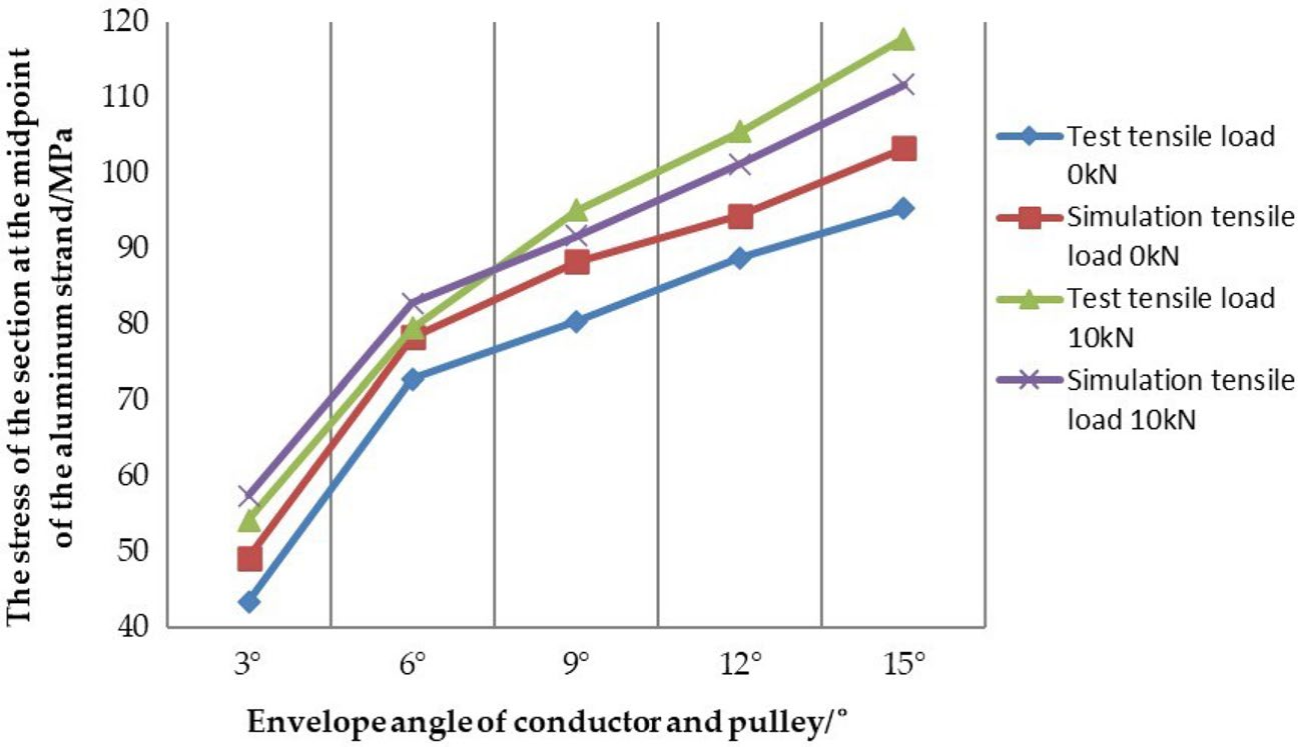
Comparison of stress data under different working conditions.

In order to further study the stress characteristics of aluminum strand under different working conditions when the conductor passes the pulley, and preliminarily verify the correctness and rationality of the same layer strand model. Now, the data obtained from the simulation condition 1, the test condition 1, the simulation condition 2 and the test condition 2 is shown in Table 13 and Table 14, and the curve diagram of the section at the midpoint of the aluminum strand with the change of the envelope angle is shown in Fig. 21 under the test condition 1, the test condition 2, the simulation condition 1 and the simulation condition 2.

**Table 13:** Data comparison between the simulation condition 1 and the test condition 1.

Envelope angle between conductor and pulley/°	3°	6°	9°	12°	15°
Section stress under test condition 1/MPa	43.496	72.829	80.483	88.825	95.287
Section stress under simulation condition 1/MPa	49.253	78.357	88.348	94.403	103.338
Error of simulation and test/%	13.236	7.590	9.772	6.280	8.449

**Table 14:** Data comparison between the simulation condition 2 and the test condition 2.

Envelope angle between conductor and pulley/°	3°	6°	9°	12°	15°
Section stress under test condition 2/MPa	54.267	79.567	95.150	105.554	117.838
Section stress under simulation condition 2/MPa	57.429	82.857	91.707	101.189	111.718
Error of simulation and test/%	5.827	4.135	3.618	4.135	5.194

In Table 13, except for the condition of envelope angle of 3°, the error of simulation and test is more than 10%, the error of other conditions is within 10%. In Table 14, comparing the data of the simulation condition 2 and the test condition 2, all errors are about 5%. The reasons for this error may be due to the accuracy of the instrument itself, and some errors in the measurement, errors in the data acquisition process and the accuracy of the numerical simulation analysis. However, it can be known that such errors are within the acceptable range. Therefore, on the whole, the stress value of the section at the midpoint of the aluminum strand obtained by the numerical simulation is in good agreement with the value obtained by the test, which also shows that the conductor model used in the numerical simulation is relatively correct and reasonable. In Fig. 21, it can also be seen that the numerical simulation results are in good agreement with the test results under the two conditions. Whether it is the trend in the drawing or the comparative analysis of simulation and test, it can preliminarily prove that the same layer strand model proposed in this paper has better correctness and rationality, which also paves the way for our further research on the damage mechanism of the conductor in the future.

In addition, it can be seen from the tables and the curve diagram under the two working conditions that the envelope angle of the conductor and the pulley and the tension load of the conductor are the two main factors affecting the internal stress of the aluminum strand, and the influence of envelope angle on the stress value of aluminum strand is more obvious than that of tension load. Therefore, in the construction of tension setting out, it is necessary to take good protection measures for the conductor as a whole, and to minimize the envelope angle between the conductor and the pulley within the allowable range. In addition, considering the influence of the pulley on the aluminum strand, the reasonable type of paying off pulley and the elastic washer should be placed at the contact position between the conductor and the wheel groove to reduce the damage of the pulley on the aluminum strand.

## Conclusions

In order to discuss the inter strand stress characteristics of the aluminum strands in the bending state when the conductor passes through the pulley, the same layer strand model and the adjacent layer strand model based on related theories and technologies are constructed. Then through the comparison and analysis of the test and the simulation, the stress characteristics of the conductor in the bending state are investigated and the rationality of the model is preliminarily verified.

The following conclusions can be drawn from this study:

1. The equivalent stress (von-Mises stress) in the cross section of aluminum strands shows a decreasing trend from the edge position to the neutral layer, and the maximum equivalent stress appears at the contact position of the adjacent layer strands.
2. The larger the envelope angle and tension load of the aluminum strands, the greater the equivalent stress in the cross section. Moreover, relative to the envelope angle, the tension load has an appropriately small effect on the aluminum strands. The friction between the aluminum strands has a certain effect on reducing the equivalent stress in the cross section.
3. The equivalent stress of aluminum strands increases from the outer layer to the inner layer. The inner aluminum strands are more likely to be damaged than the outer aluminum strands.
4. After comparing and analyzing the test results and the simulation results, it is found that the equivalent stress at the midpoint of the aluminum strand obtained by the numerical simulation is in good agreement with the equivalent stress obtained in the test, which proves that the same layer strand model used in the numerical simulation is more accurate and reasonable. In addition, the influence of the envelope angle on the equivalent stress of the aluminum strand is more obvious than that of the tension load. Therefore, during the construction of tension setting out, it is necessary to take protective measures for the conductor, and reduce the envelope angle between conductor and pulley as much as possible within the allowable range.
